# Supplementary figures and images for: Hexokinase 2 is an RNA-binding protein that regulates mRNA translation independently of glycolysis and induces melanoma cell proliferation
Source: PLoS Biol. 2025 Sep 16;23(9):e3003364. doi: 10.1371/journal.pbio.3003364 (PMC12494293; doi:10.1371/journal.pbio.3003364)

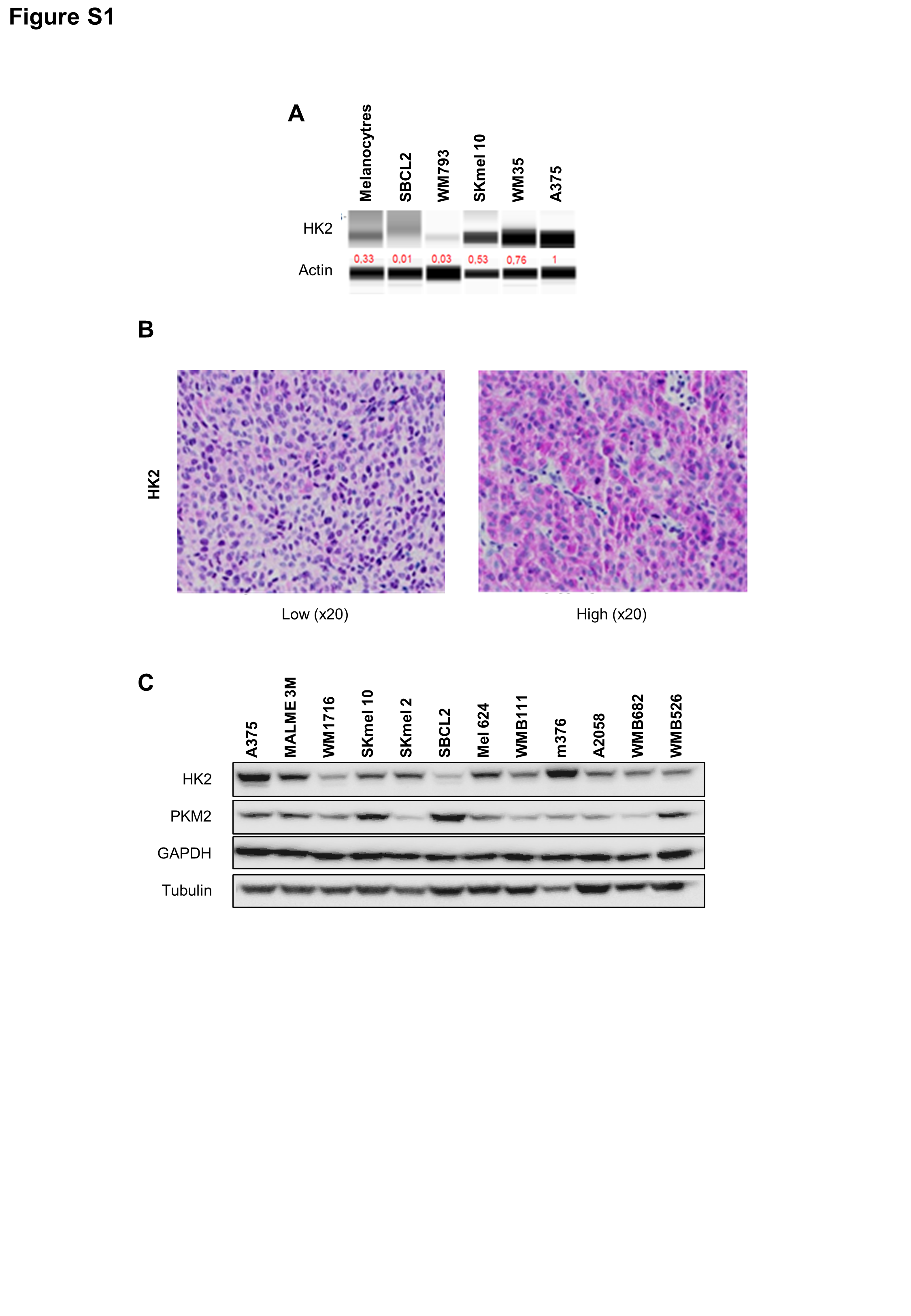

Supplement: S1 Fig — (A) HK2 expression level assessed by microfluidic western blot analysis of normal melanocytes, early superficial melanoma with radial growth (RGP, SBCL2), early invasive melanoma (VGP, WM793), low invasive (SKMel10) and high invasive (A375) metastatic melanoma cell lines. Actin was used as loading control. Quantification of HK2 protein levels are normalized to actin expression and presented in red, relative to HK2 expression in A375 cells. (B) Immunohistochemical analysis of HK2 expression in three representative samples from a cohort of 31 patients with cutaneous melanoma. Left: representative melanoma sample with low HK2 expression. Right: representative melanoma sample with high HK2 expression. (C) Western blot analysis of key glycolytic enzymes (HK2, PKM2 and GAPDH) in a variety of melanoma cell lines. Tubulin was used as loading control. (TIF) [file pbio.3003364.s001.TIF]

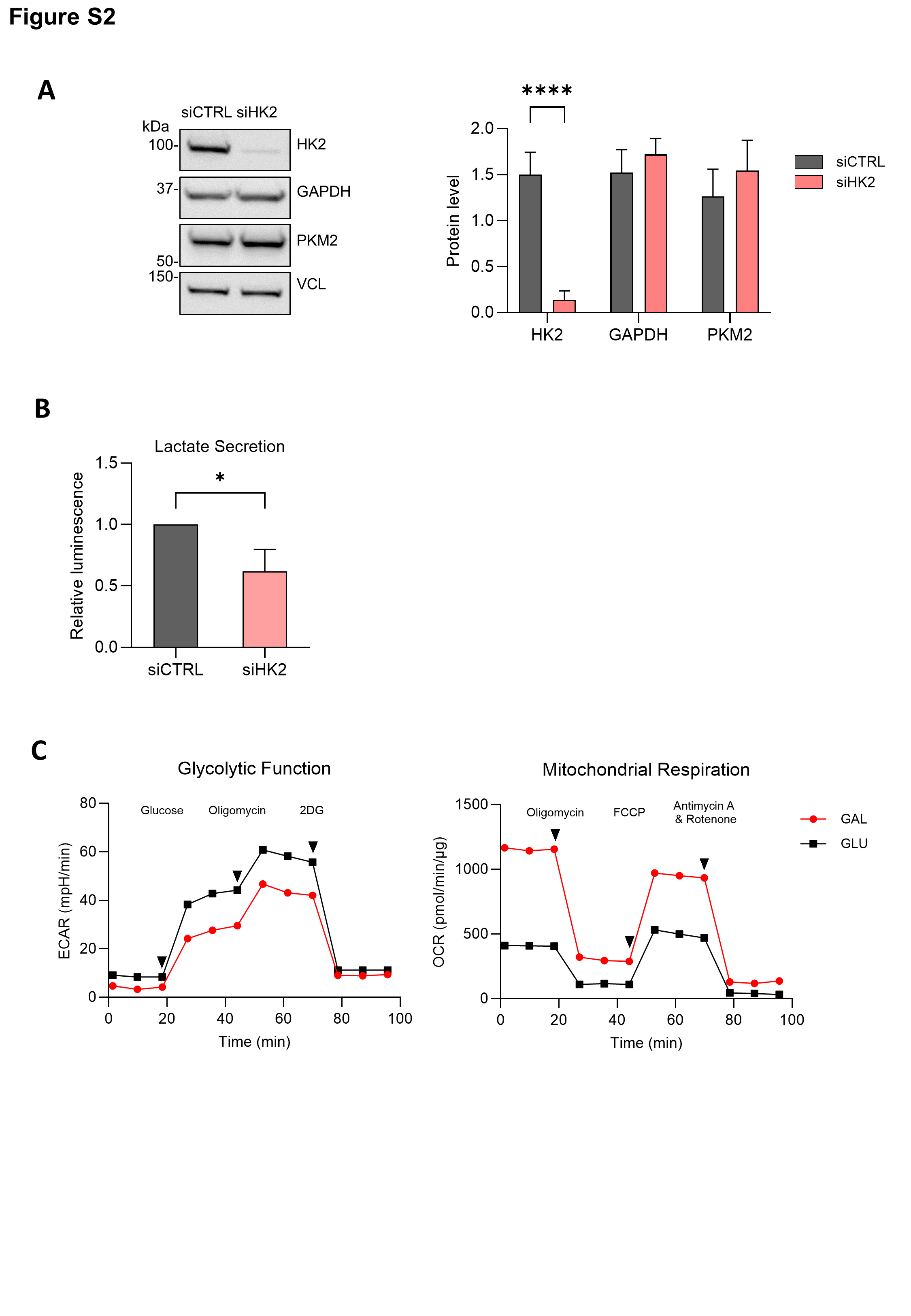

Supplement: S2 Fig — (A) (Left panel) western blot analysis of HK2, GAPDH, and PKM2 protein levels in A375 melanoma cells upon siRNA-mediated depletion of HK2 (siHK2, light red) or control (siCTRL, gray). VCL was used as loading control. (Right panel) Quantification of HK2, GAPDH, and PKM2 protein levels are normalized to VCL expression. p-values were calculated by ordinary two-way ANOVA with Šídák’s multiple comparisons test (SD, n = 3 biological replicates) and only significant comparisons are shown (** p ≤ 0.01, *** p ≤ 0.001). (B) Measurement of lactate secretion in A375 cells in A375 melanoma cells upon siRNA-mediated depletion of HK2 (siHK2, light red) or control (siCTRL, gray). p-values were calculated by unpaired, two-tailed Student t test test (SD, n = 3 biological replicates) and only significant comparisons are shown (* p ≤ 0.05). (C) Right panel: the extra-cellular acidification rate (ECAR), an indicator of aerobic glycolysis, was measured in A375 cells grown in either glucose or galactose-containing medium followed by consecutive treatments of glucose (glc), oligomycin, and the HK2 inhibitor 2-DG (n = 1). Left panel: the oxygen consumption rate (OCR) was measured in A375 cells cultured either in glucose or galactose-containing medium followed by consecutive treatments of oligomycin, FCCP, and antimycin A and rotenone (n = 1). The individual numerical values for panels S2A–S2C Fig are available at S8 Data. (TIF) [file pbio.3003364.s002.TIF]

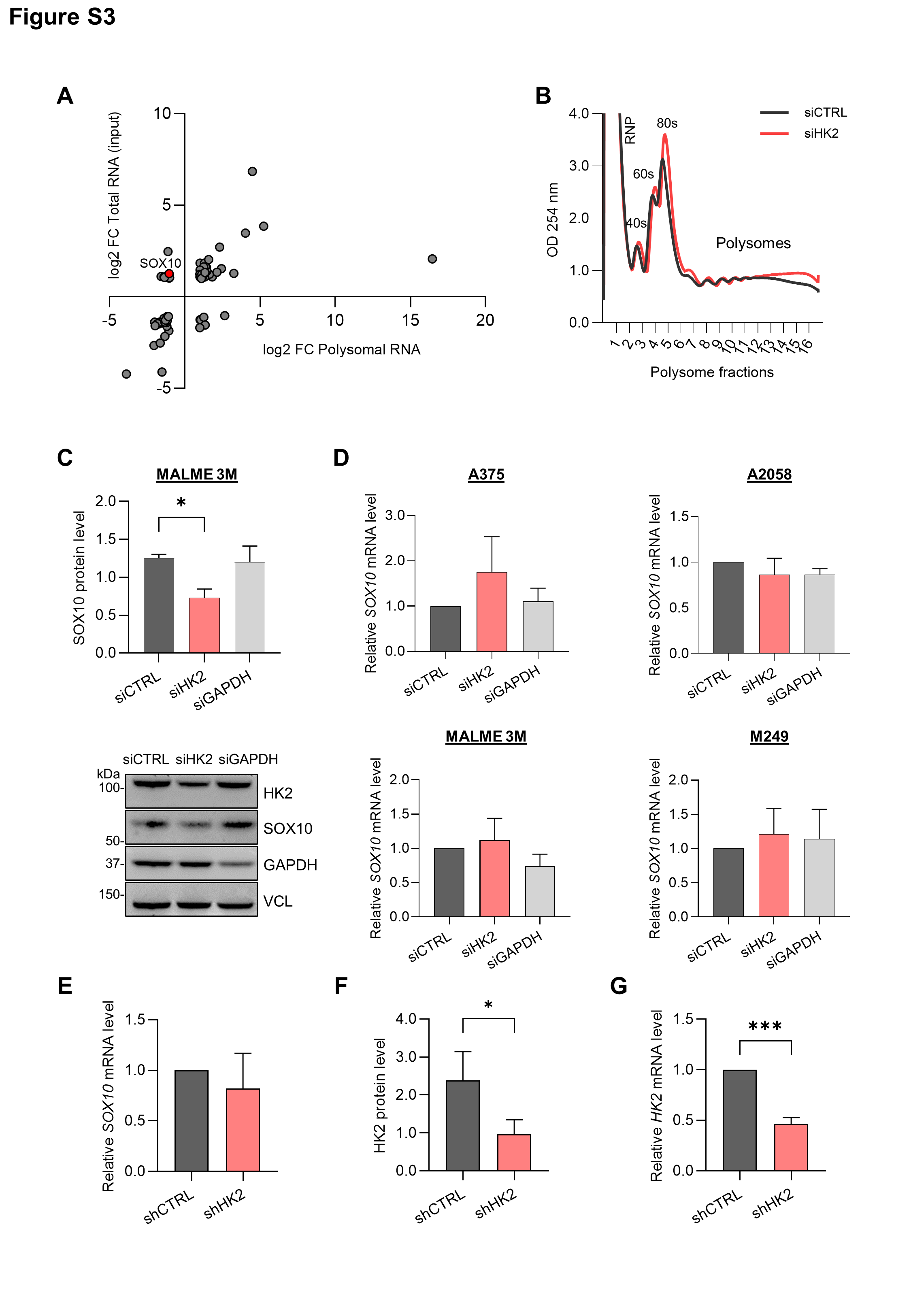

Supplement: S3 Fig — (A) RT-qPCR quantification of 84 EMT-associates genes. Transcriptional (total RNA) and translational (polysomal RNA) levels of each gene were obtained from polysome profiling of A375 cells transfected with siRNAs targeting HK2 (siHK2) or control (siCTR) and measured using RT2 Profiler PCR Array (N = 1). (B) Polysome profile of A375 cells upon siRNA-mediated depletion of HK2 (siHK2, light red) or control (siCTRL, gray), assessed by sucrose-gradient (10%–50%) ultracentrifugation. (C) western blot analysis of the SOX10 protein level in MALME 3M melanoma cell line transfected with siRNAs targeting HK2 (siHK2, light red), GAPDH (siGAPDH, light gray) or control (siCTRL, gray). The SOX10 protein quantification is normalized to VCL expression. p-values were calculated by two-tailed unpaired t test (SD, n = 3 biological replicates) and only significant comparisons are shown (* p ≤ 0.05). (D) RT-qPCR quantification of the SOX10 mRNA levels in different melanoma cell lines transfected with siRNAs targeting HK2 (siHK2, light red), GAPDH (siGAPDH, light gray) or control (siCTRL, gray). p-values were calculated by ordinary one-way ANOVA (SD, n = 3 biological replicates). (E) RT-qPCR quantification of the SOX10 mRNA level in A375 cells upon stable HK2 knockdown. p-values were calculated by two-tailed unpaired t test (SD, n = 3 biological replicates). (F) western blot quantification of the HK2 protein level in A375 cells upon stable HK2 knockdown. HK2 protein levels are normalized to VCL expression. p-value was calculated by two-tailed unpaired t test (SD, n = 3 biological replicates) (* p ≤ 0.05). (G) RT-qPCR quantification of the HK2 mRNA level in A375 cells upon stable HK2 knockdown. p-values were calculated by two-tailed unpaired t test (SD, n = 3 biological replicates) (*** p ≤ 0.001). The individual numerical values for panels S3A–S3G Fig are available at S9 Data. (TIF) [file pbio.3003364.s003.TIF]

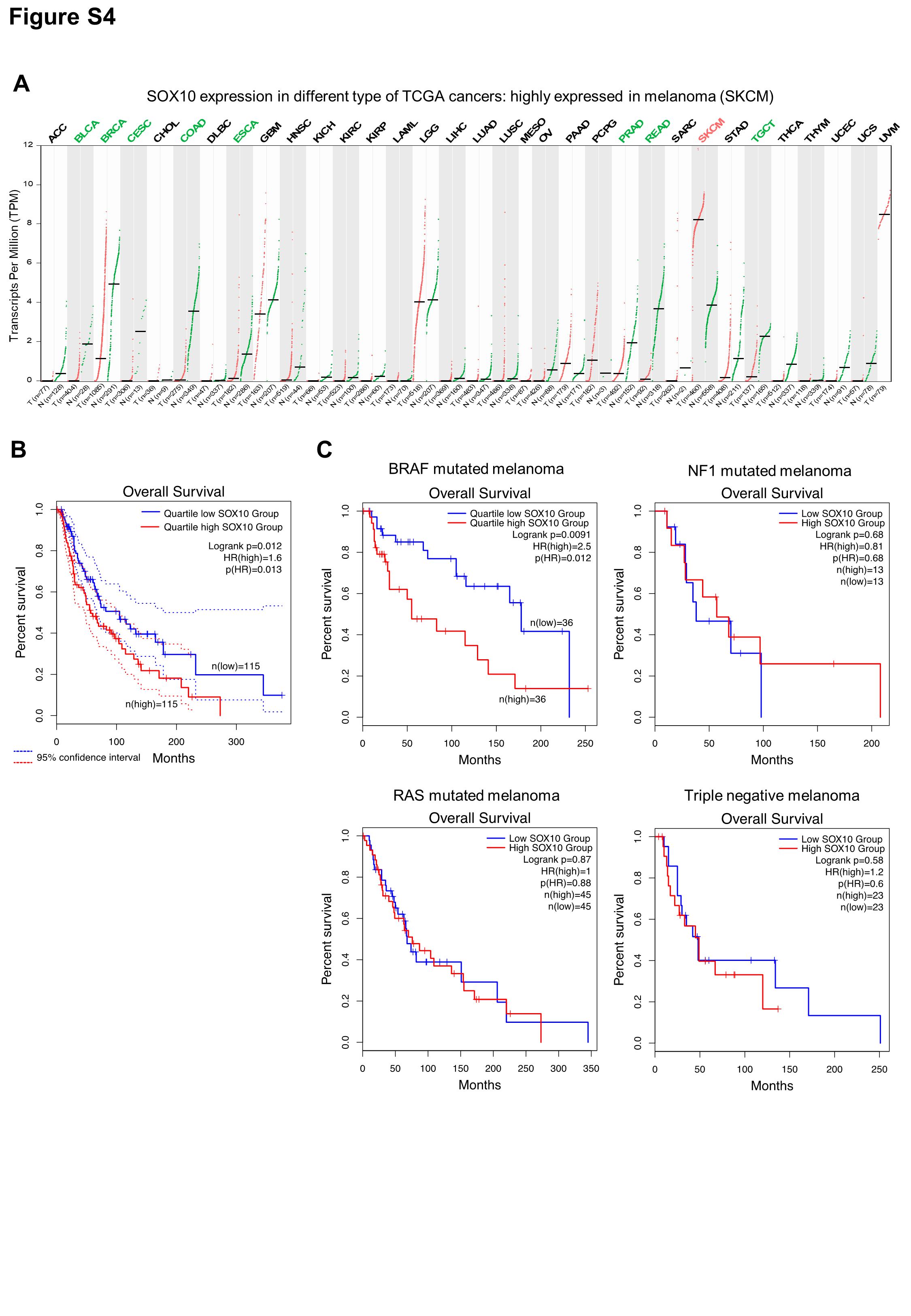

Supplement: S4 Fig — (A) SOX10 expression in different type of solid cancers from TGCA database (https://www.cancer.gov/ccg/research/genome-sequencing/tcga). T: tumor (red dots); N: normal tissue (green dots). (B) Analysis of the impact of SOX10 expression in overall survival of patients with cutaneous melanoma. (C) Analysis of the impact of SOX10 expression in overall survival according to molecular profile of the tumor. See Materials and methods for information on how the graphs were generated. (TIF) [file pbio.3003364.s004.TIF]

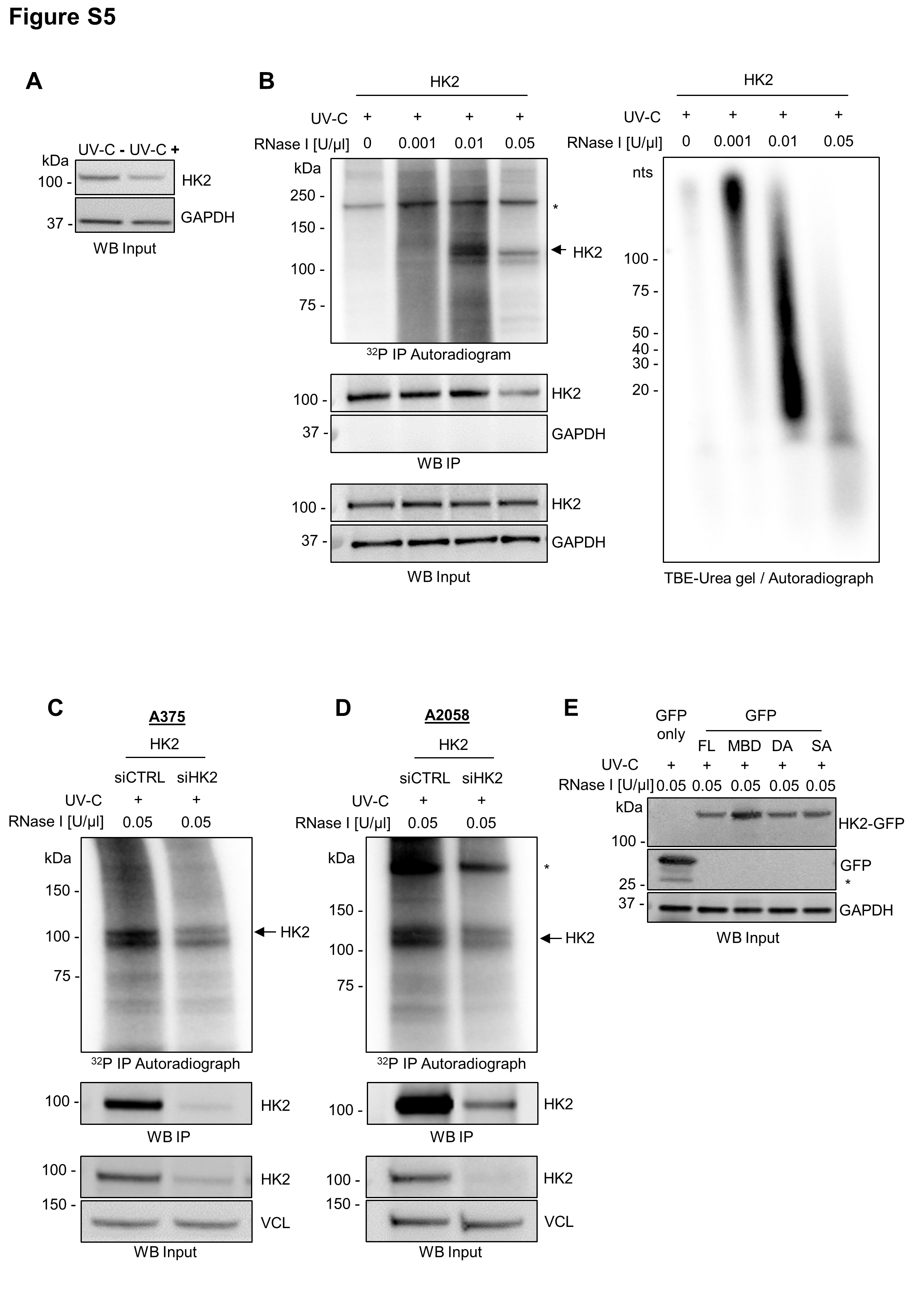

Supplement: S5 Fig — (A) Western blots of HK2 and GAPDH (normalization control) input of the CLIP from endogenous HK2 in A375 cells presented in Fig 3B. (B) CLIP from endogenous HK2 in A375 cells (n = 3 biological replicates). Left upper panel: autoradiography of HK2-RNA complexes in UV-C treated (+) A375 cells upon increasing concentrations of RNase I. One major band is observed in the expected molecular mass of HK2, as indicated by a black arrow, and the asterisk (*) indicates a nonspecific band. Left middle and lower panel: western blots of HK2 and IgG immunoprecipitation, and HK2 and GAPDH (normalization control) input, respectively. Same conditions as the upper panel. Right panel: autoradiography of RNA purified from 32P labeled RNA-HK2 complexes (observed in the left upper panel), migrated on a denaturing TBE-urea gel. The purified RNA migrates as a smear, and it is sensitive to increasing concentrations of RNase I. (C) CLIP from endogenous HK2 in A375 cells upon siRNA-mediated depletion of HK2 (siHK2) in comparison to control (siCTRL) (n = 3 biological replicates). Upper panel: autoradiography of HK2-RNA complexes in UV-C (+) and RNase I treated (0.05 U/μL) A375 cells. One major band is observed in the expected molecular mass of HK2, as indicated by a black a3rrow. Left middle and lower panel: western blots of HK2 and IgG immunoprecipitation, and HK2 and GAPDH (normalization control) input, respectively. Same conditions as the upper panel. (D) CLIP from endogenous HK2 in A2058 cells upon siRNA-mediated depletion of HK2 (siHK2) in comparison to control (siCTRL) (n = 3 biological replicates), same conditions as in (C). One major band is observed in the expected molecular mass of HK2, as indicated by a black arrow, and the asterisk (*) indicates a nonspecific band. (E) Western blots of GFP input of the CLIP from GFP-only or GFP-HK2 transfected HEK293T cells presented in Fig 3C. The arrow indicates the expected HK2-GFP molecular mass, and the asterisk (*) indicates a nonspecific band [file pbio.3003364.s005.TIF]

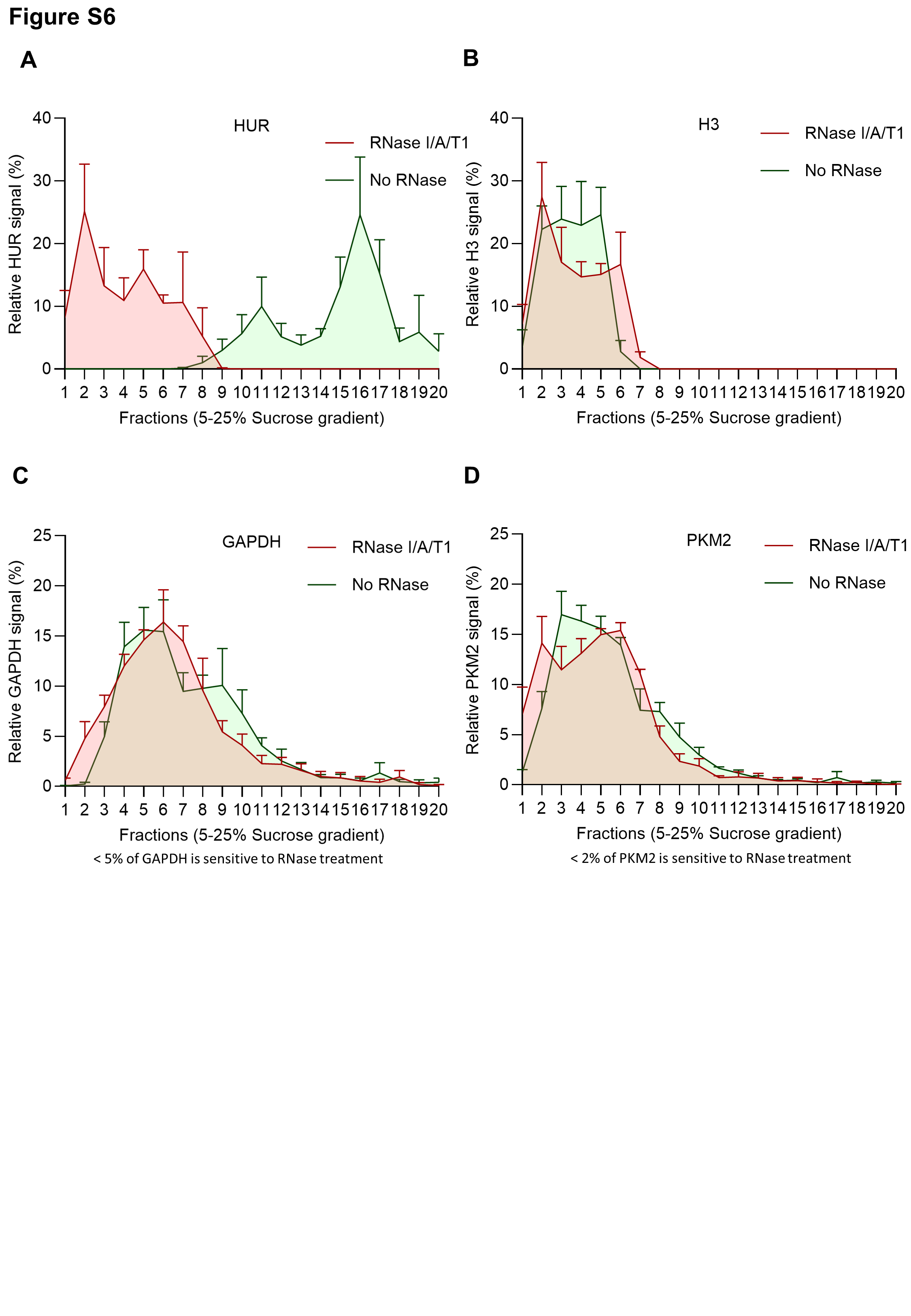

Supplement: S6 Fig — Western blot quantification representing the % of (A) HUR, (B) H3, (C) GAPDH, and (D) PKM2 in each sucrose fraction of lysates treated with RNase I/A/T1 or left untreated (SD, n = 4 biological replicates). The individual numerical values for panels S6A–S6D Fig are available at S10 Data. (TIF) [file pbio.3003364.s006.TIF]

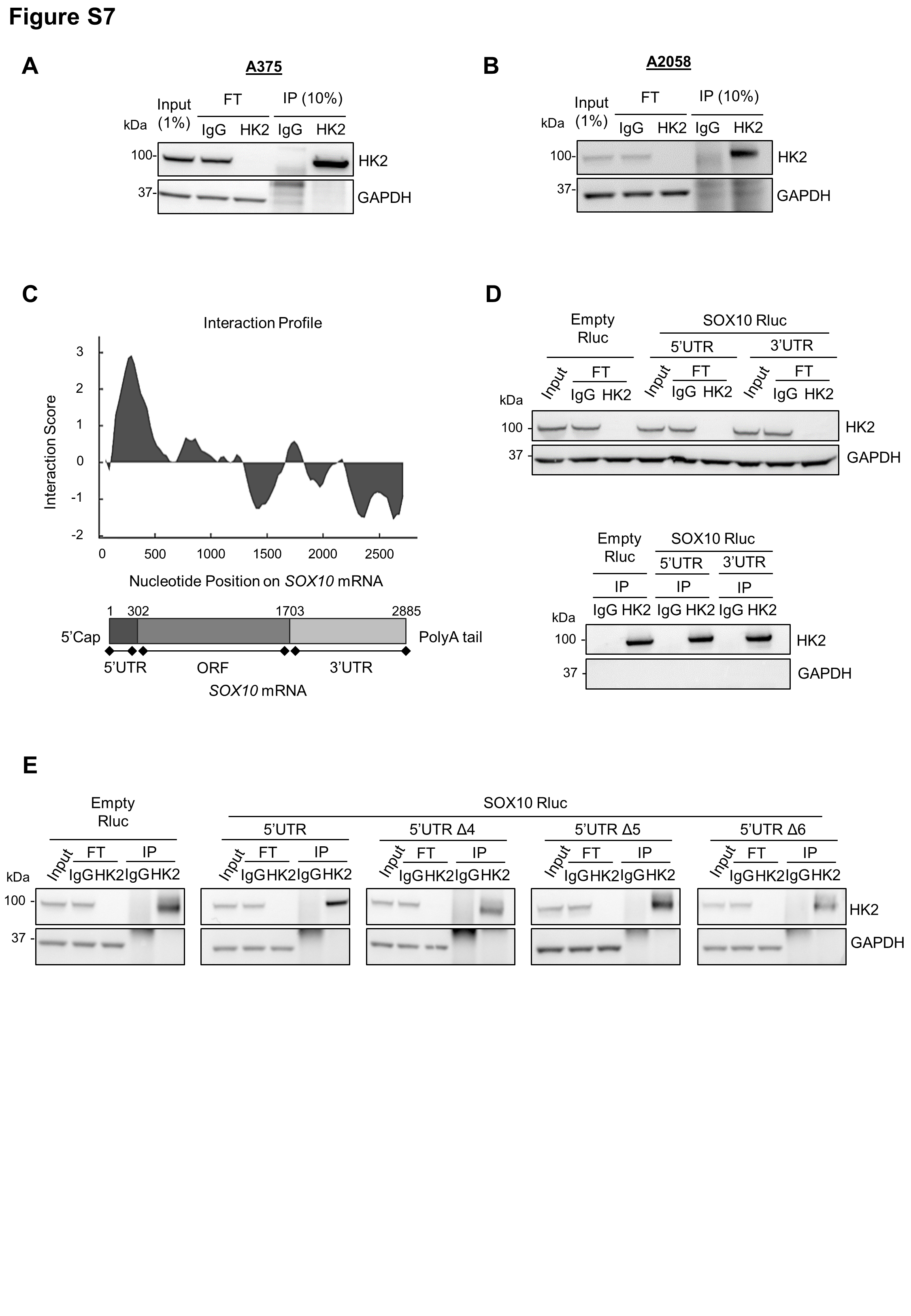

Supplement: S7 Fig — (A) western blot of HK2 and GAPDH (normalization control) from RIP experiment performed on A375 and (B) A2058 melanoma cell lines (representative images, n = 3 biological replicates). (C) In silico prediction of HK2-SOX10 mRNA interaction propensities using the CatRAPID algorithm. The highest interaction score observed between HK2 and the SOX10 mRNA is at the SOX10 5′UTR. Upper panel: HK2-SOX10 mRNA interaction profile. Lower panel: schematic representation of the SOX10 mRNA. (D) western blot of HK2 and GAPDH (normalization control) from RIP experiment performed on A375 cells transfected with luciferase reporters containing the SOX10 5′UTR and 3′UTR sequences upstream of the RLuc luciferase reporter gene. An empty reporter was used as control (representative images, n = 3 biological replicates). (E) western blot of HK2 and GAPDH (normalization control) from RIP experiment performed on A375 cells transfected with luciferase reporters containing the SOX10 5′UTR and the Δ4, Δ5, and Δ6 deletion mutant sequences upstream of the RLuc luciferase reporter gene. An empty reporter was used as control (representative images, n = 3 biological replicates). The individual numerical values for panels S7C Fig are available at S11 Data. (TIF) [file pbio.3003364.s007.TIF]

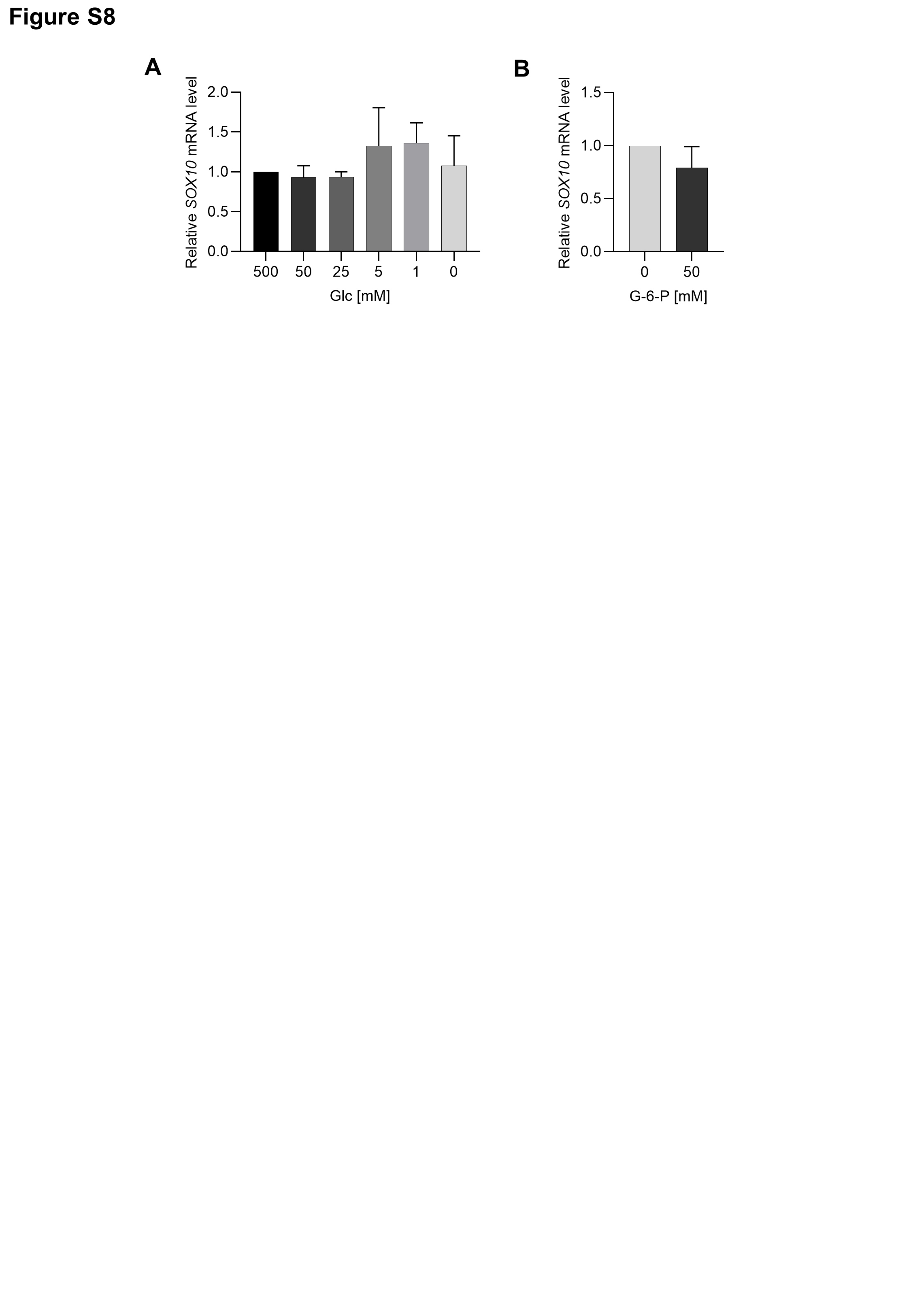

Supplement: S8 Fig — (A) RT-qPCR quantification of the SOX10 mRNA level in A375 cells cultured for 24 h with decreasing concentrations of glucose or under glucose starvation, as indicated (SD, n = 3 biological replicates). (B) RT-qPCR quantification of the SOX10 mRNA level in A375 cells cultured in media without glucose supplemented or not with G-6-P (50 mM) for 24 h (SD, n = 3 biological replicates). The individual numerical values for panels S8A, S8B Fig are available at S12 Data. (TIF) [file pbio.3003364.s008.TIF]

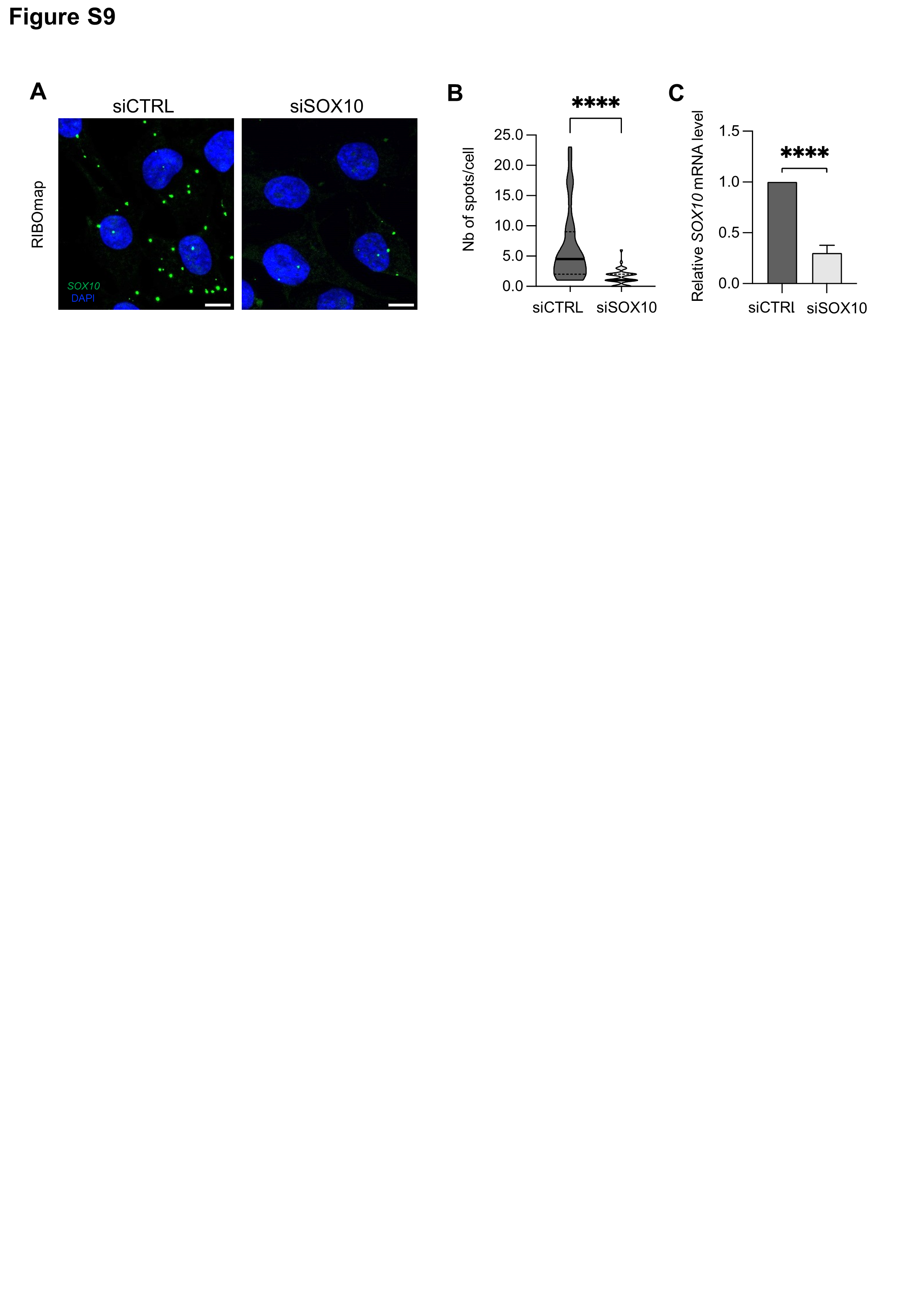

Supplement: S9 Fig — (A) Representative confocal images of translating SOX10 mRNAs (green spots) detected by RIBOmap assay in A375 cells transfected with control siRNA (siCTRL) or with siRNA targeting SOX10 (siSOX10). Nuclei are stained with DAPI (blue). Scale bar: 10 µm. (B) Quantification of the RIBOmap signal in the condition described in (A). The data shown represent the number of spots/cells from a representative experiment (n = 3 biological replicates). p-values were calculated by unpaired, two-tailed Student t test (**** p < 0.0001). (C) RT-qPCR quantification of SOX10 mRNA level in the condition described in g. mean ± SD, n = 3 biological replicates. p-values were calculated by unpaired, two-tailed Student t test (**** p < 0.0001). The individual numerical values for panels S9B Fig are available at S13 Data. (TIF) [file pbio.3003364.s009.TIF]

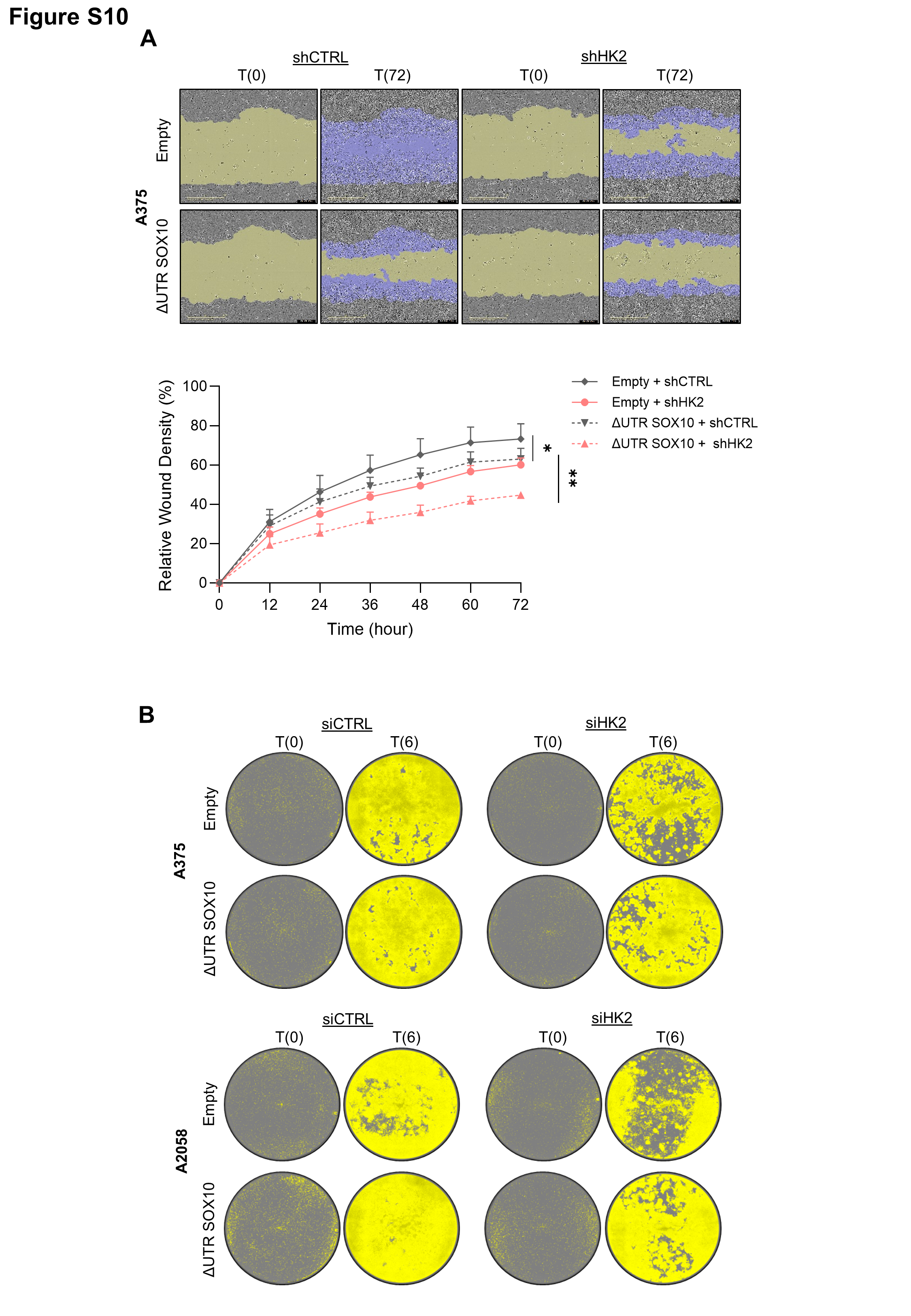

Supplement: S10 Fig — (A) Scratch-wound assay comparing the migratory capacity of A375 cells upon stable HK2 knockdown (shHK2, light red) to parental cells (shCTRL, gray), ectopically expressing or not SOX10 (ΔUTR SOX10—dashed lines—and Empty, respectively). Upper panel: representative images of the wounded areas of the first acquisition at time zero (T(0)), and at the last acquisition after 72 h (T(72)). Scratch wound area is shown in yellow, initial scratch wound covered by the cells is shown in blue, and the cells adjacent to the initial scratch wound are shown in gray. Lower panel: Relative Wound Density (%) was used to report the data, which was calculated by measuring the spatial cell density in the wounded area relative to the density outside of the same area at the indicated time points. Cells and gap areas were photographed every 3 h for 72 h. Significance was calculated by comparing HK2 knockdown to parental cells after 72 h. p-values were calculated by ordinary two-way ANOVA with Turkey’s multiple comparison test (SD, n = 3 biological replicates), and only significant differences within the same cell line are shown (*p ≤ 0.05; ** p ≤ 0.01). (B) Proliferation assay comparing the percent of plate coverage (cell confluence) of A375 or A2058 cells upon siRNA-mediated depletion of HK2 (shHK2) to parental cells (shCTRL), ectopically expressing or not SOX10 (ΔUTR SOX10 and Empty). Upper panel: representative images of the cell confluence of A375 cells at the moment of the first acquisition at day zero (T(0)), and at the last acquisition after 6 days (T(6)) (n = 3 biological replicates, quantifications in the left panel of Fig 7D). Lower panel: representative images of the cell confluence of A2058 cells at the moment of the first acquisition at day zero (T(0)), and at the last acquisition after 6 days (T(6)) (n = 3 biological replicates, quantifications in the right panel of Fig 7D). Area not covered by the cells is shown in gray, while covered area is shown in yellow. The individual [file pbio.3003364.s010.TIF]

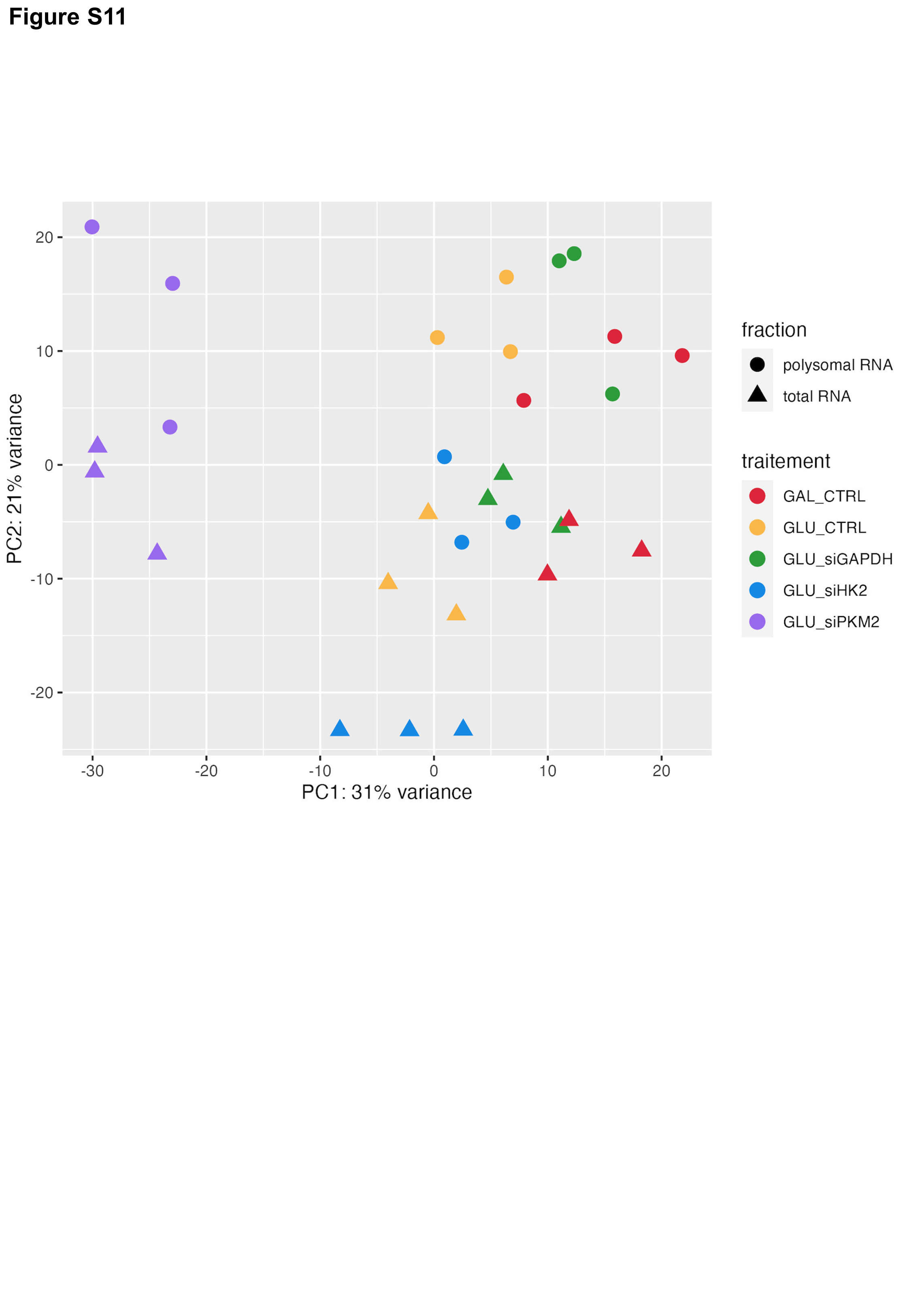

Supplement: S11 Fig — The VST offered by DESeq2 was used on the raw count data to stabilize the variance across the mean. The principal components analysis (PCA) plot was built using the ggplot2 package. The individual numerical values for panels S11 Fig are available at S15 Data. (TIF) [file pbio.3003364.s011.TIF]
