# Supplementary material for: Hexokinase 2 is an RNA-binding protein that regulates mRNA translation independently of glycolysis and induces melanoma cell proliferation
Source: PLoS Biol. 2025 Sep 16;23(9):e3003364. doi: 10.1371/journal.pbio.3003364 (PMC12494293; doi:10.1371/journal.pbio.3003364)
Supplement: S3 Table — RT-qPCR primers, EMSA RNA oligonucleotides, PLA and RIBOmap probes. (DOCX) [file pbio.3003364.s014.docx]

**S3 Table. Relevant oligonucleotides.**

| **NAME OF THE OLIGONUCLEOTIDE** | **SEQUENCE** |
| --- | --- |
| **RT-qPCR primers** |  |
| VCL Forward | TGATGATTAGAGACATCACCGCT |
| VCL Reverse | AACAAAGGAGAAACCTGACCT |
| GAPDH Forward | TCCCATCACCATCTTCCAGG |
| GAPDH Reverse | TCCATGGTGGTGAAGACGC |
| TBP Forward | GGAAGGGGCATTATTTGTG |
| TBP Reverse | GCCCAGATAGCAGCACGGTA |
| ACT Forward | CCGTGTTTCCTTCCATCGTC |
| ACT Reverse | ACGATGCCATGCTCAATGGG |
| Renilla Forward | ACGGATGATAACTGGTCCGC |
| Renilla Reverse | CGCGCTACTGGCTCAATATG |
| Firefly Forward | GAAATGTCCGTTCGGTTGGC |
| Firefly Reverse | TCCGATAAATAACGCGCCCA |
| OCLN Forward | GGTCGGGCCCAGTTGC |
| OCLN Reverse | ATGATTCGGTTTGAATTCATCAGG |
| CDH2 Forward | ACTCCAGGGGACCTTTTCCT |
| CDH2 Reverse | TGCCCTCAAATGAAACCGGG |
| PTK2 Forward | TTGGGCGGAAAGAAATCCTG |
| PTK2 Reverse | GTCCAGGTTGGCAGTAGGAG |
| TMP1 Forward | GACACCAGAGAACCCACCAT |
| TMP1 Reverse | CACGAACTTGGCCCTGATGA |
| WNT5A Forward | GCTCGCATCCTCATGAACCT |
| WNT5A Reverse | GCCACATCAGCCAGGTTGTA |
| SOX10 5'UTR Forward | CACTTCCTAAGGACGAGCCC |
| SOX10 5'UTR Reverse | TCCTCGCAAAGAGTCCAACG |
| SOX10 CDS Forward | GGCTGCTGAACGAAAGTGA |
| SOX10 CDS Reverse | TCTTGTAGTGGGCCTGGATG |
|  |  |
| **EMSA** |  |
| ATTO700 *SOX10* stem-loop | AGGACGAGCCCCAGACUGGAGGAGAGGUCC |
| *SOX10* stem-loop | AGGACGAGCCCCAGACUGGAGGAGAGGUCC |
| *SOX10* mutated stem-loop | AGGACGAGCCCCUCUGAGGAGGAGAGGUCC |
|  |  |
| **PLA** |  |
| MINUS probe (*SOX10* 5’UTR) | GCGGTCCAGCTCGGGGCTGGGAGGTGACGCTGGTGGGCTGGGAGGGAAAAAAAAAAAAAAAAAAAAATATGACAGAACTAGACACTCTT |
|  |  |
| **RIBOmap** |  |
| Splint_18SRNA_50A_InvdT_1 | ACAAAATAGAACCGCGGTCCTATTCAA AAA AAA AAA AAA AAA AAA AAA AAA AAA AAA AAA AAA AAA AAA AAA AAA TAT CTT TAG T*G*T* /3InvdT/ |
| Splint_18SRNA_50A_InvdT_2 | CATCGTTTATGGTCGGAACTACGACAA AAA AAA AAA AAA AAA AAA AAA AAA AAA AAA AAA AAA AAA AAA AAA AAA TAT CTT TAG T*G*T* /3InvdT/ |
| Splint_18SRNA_50A_InvdT_3 | AGGTTTCCCGTGTTGAGTCAAATTAA AAA AAA AAA AAA AAA AAA AAA AAA AAA AAA AAA AAA AAA AAA AAA AAA TAT CTT TAG T*G*T* /3InvdT/ |
| Splint_18SRNA_50A_InvdT_4 | TGTTATTGCTCAATCTCGGGTGGCTAA AAA AAA AAA AAA AAA AAA AAA AAA AAA AAA AAA AAA AAA AAA AAA AAA TAT CTT TAG T*G*T* /3InvdT/ |
| Splint_18SRNA_50A_InvdT_5 | AGATAGTCAAGTTCGACCGTCTTCTAA AAA AAA AAA AAA AAA AAA AAA AAA AAA AAA AAA AAA AAA AAA AAA AAA TAT CTT TAG T*G*T* /3InvdT/ |
|  |  |
| SOX10_RIBOmap_padlock_1 | /5Phos/AAGATAAATTGGTCATCCTAGGGGAACTTGTCATCGTCCGCCTATTACTTGGTCATACACTA |
| SOX10_RIBOmap_padlock_2 | /5Phos/AAGATAAATTGGTCATCCTATTTTGCTGGCGCCGTTGACGATTACTTGGTCATACACTA |
| SOX10_RIBOmap_padlock_3 | /5Phos/AAGATAAATTGGTCATCCTATTGCCCGACTGCAGCTCTGTATTACTTGGTCATACACTA |
| SOX10_RIBOmap_padlock_4 | /5Phos/AAGATAAATTGGTCATCCTAGCTTCCCGCCCTCCCCCATATTACTTGGTCATACACTA |
| SOX10_RIBOmap_padlock_5 | /5Phos/AAGATAAATTGGTCATCCTACTGGCCCGAGTGGCCATAATATTACTTGGTCATACACTA |
|  |  |
| SOX10_RIBOmap primer_01 | GACGGCCTCGCGGATGCACGTGGATGACCAA |
| SOX10_RIBOmap primer_02 | GCCGCTTGACGTGCGGCTTGTGGATGACCAA |
| SOX10_RIBOmap primer_03 | CCGTCCCGCTTCGGGTCTGGTGGATGACCAA |
| SOX10_RIBOmap primer_04 | TGTCCACGTTGCCGAAGTCGATGTGTGGATGACCAA |
| SOX10_RIBOmap primer_05 | GGCCGAGTAGAGGCCAGAGGTGGATGACCAA |
|  |  |
| Detection Probe | /5Alex488N/TATTACTTGGTCATACACTA |
