# Supplementary material for: Hexokinase 2 is an RNA-binding protein that regulates mRNA translation independently of glycolysis and induces melanoma cell proliferation
Source: PLoS Biol. 2025 Sep 16;23(9):e3003364. doi: 10.1371/journal.pbio.3003364 (PMC12494293; doi:10.1371/journal.pbio.3003364)
Supplement: S1 Raw images — Uncropped blots and gels. (PDF) [file pbio.3003364.s015.pdf]

Figure 1

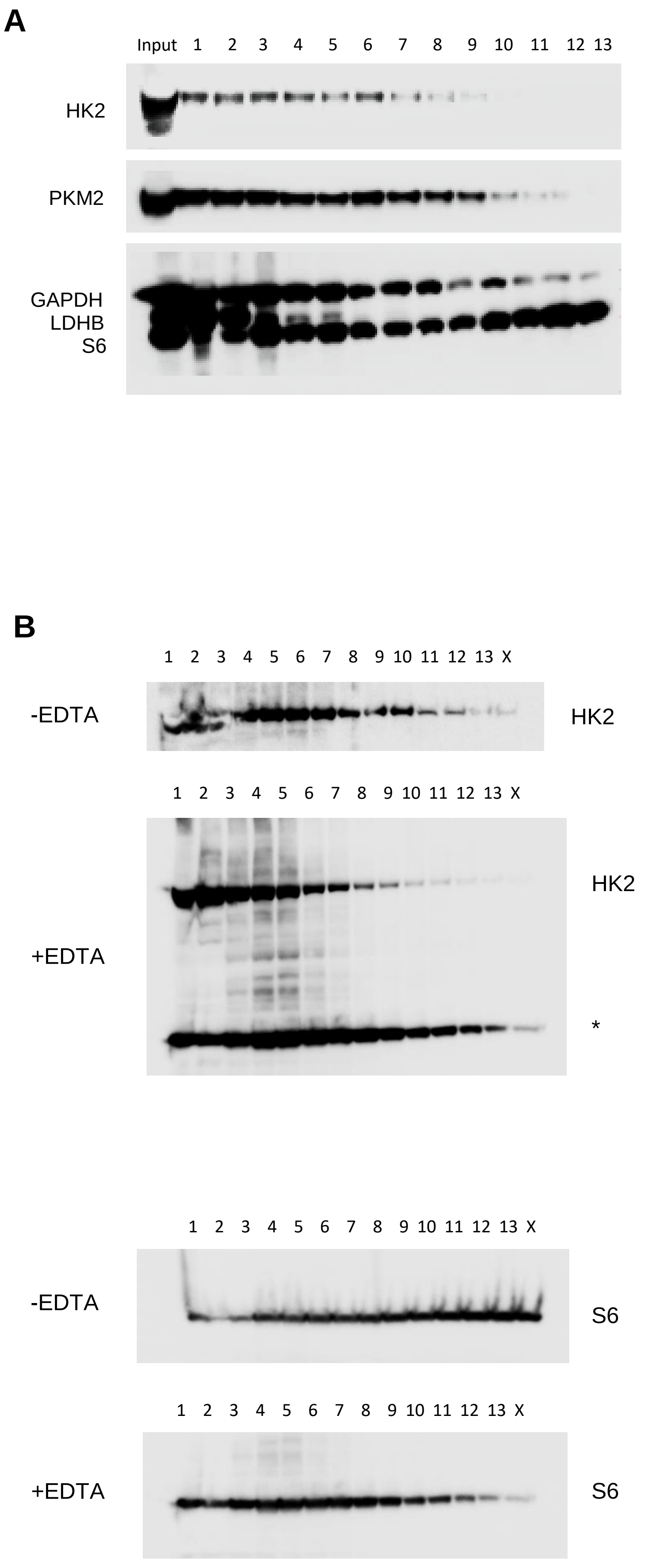
A
Input 1 2 3 4 5 6 7 8 9 10 11 12 13
HK2
PKM2
GAPDH
LDHB
S6
B
1 2 3 4 5 6 7 8 9 10 11 12 13 X
-EDTA
HK2
1 2 3 4 5 6 7 8 9 10 11 12 13 X
+EDTA
HK2
*
1 2 3 4 5 6 7 8 9 10 11 12 13 X
-EDTA
S6
1 2 3 4 5 6 7 8 9 10 11 12 13 X
+EDTA
S6

Figure 2

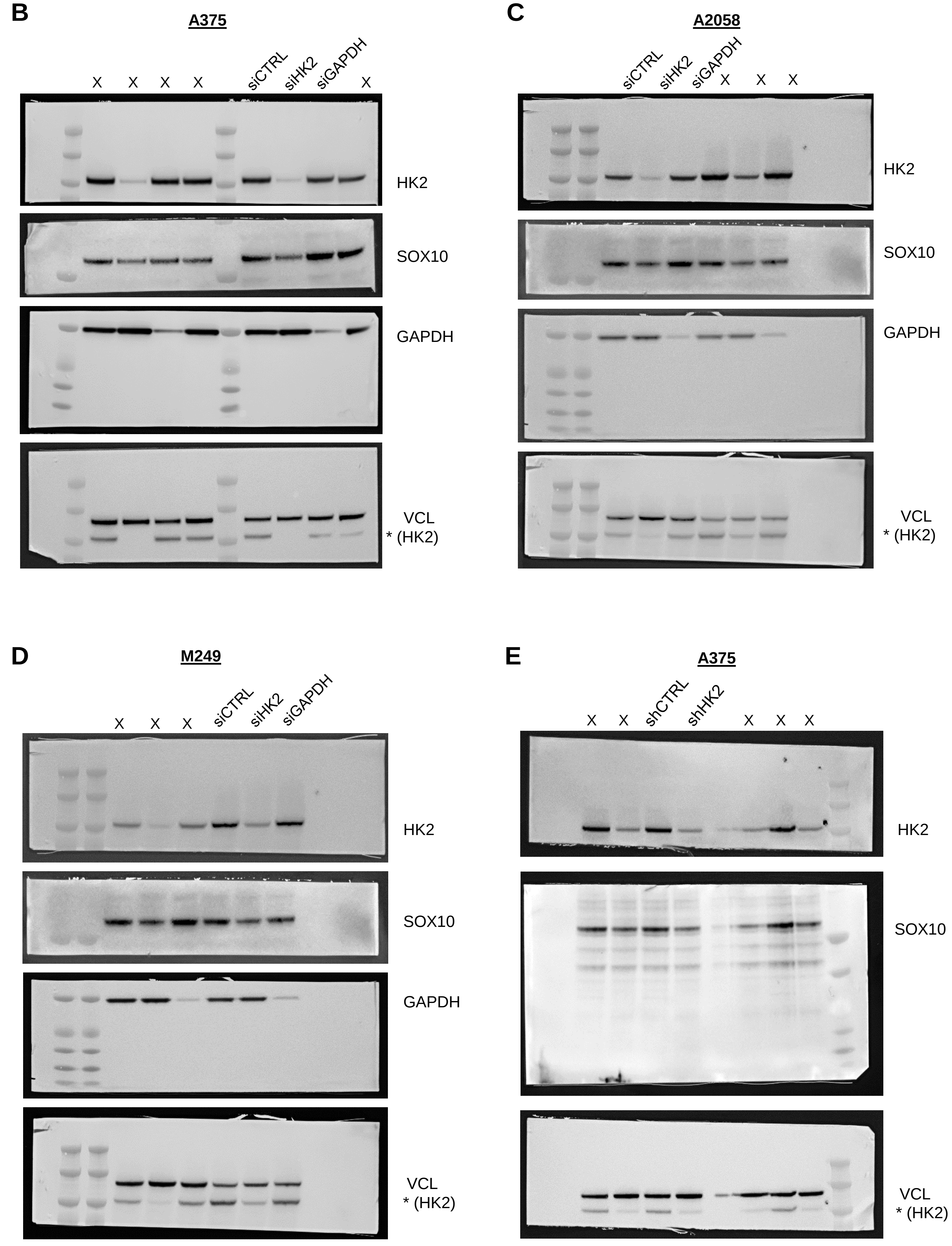
B
A375
X X X X siCTRL siHK2 siGAPDH X
HK2
SOX10
GAPDH
VCL
* (HK2)
C
A2058
siCTRL siHK2 siGAPDH X X X
HK2
SOX10
GAPDH
VCL
* (HK2)
D
M249
X X X siCTRL siHK2 siGAPDH
HK2
SOX10
GAPDH
VCL
* (HK2)
E
A375
X X shCTRL shHK2 X X X
HK2
SOX10
VCL
* (HK2)

Figure 3

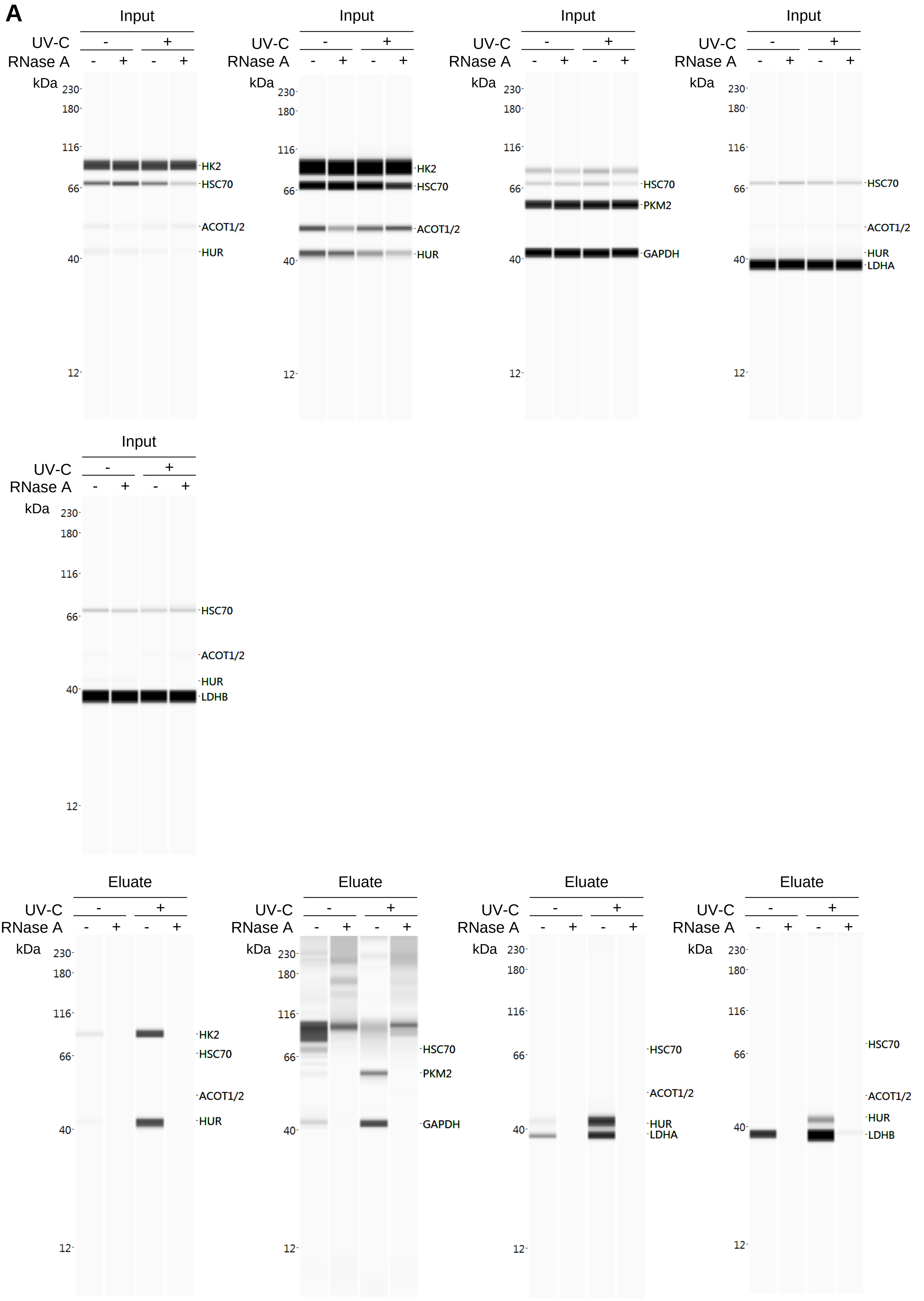

A
Input
UV-C - +
RNase A - + - +
kDa
230
180
116
66
40
12
HK2
HSC70
ACOT1/2
HUR
PKM2
GAPDH
LDHA
LDHB
Eluate

Figure 3

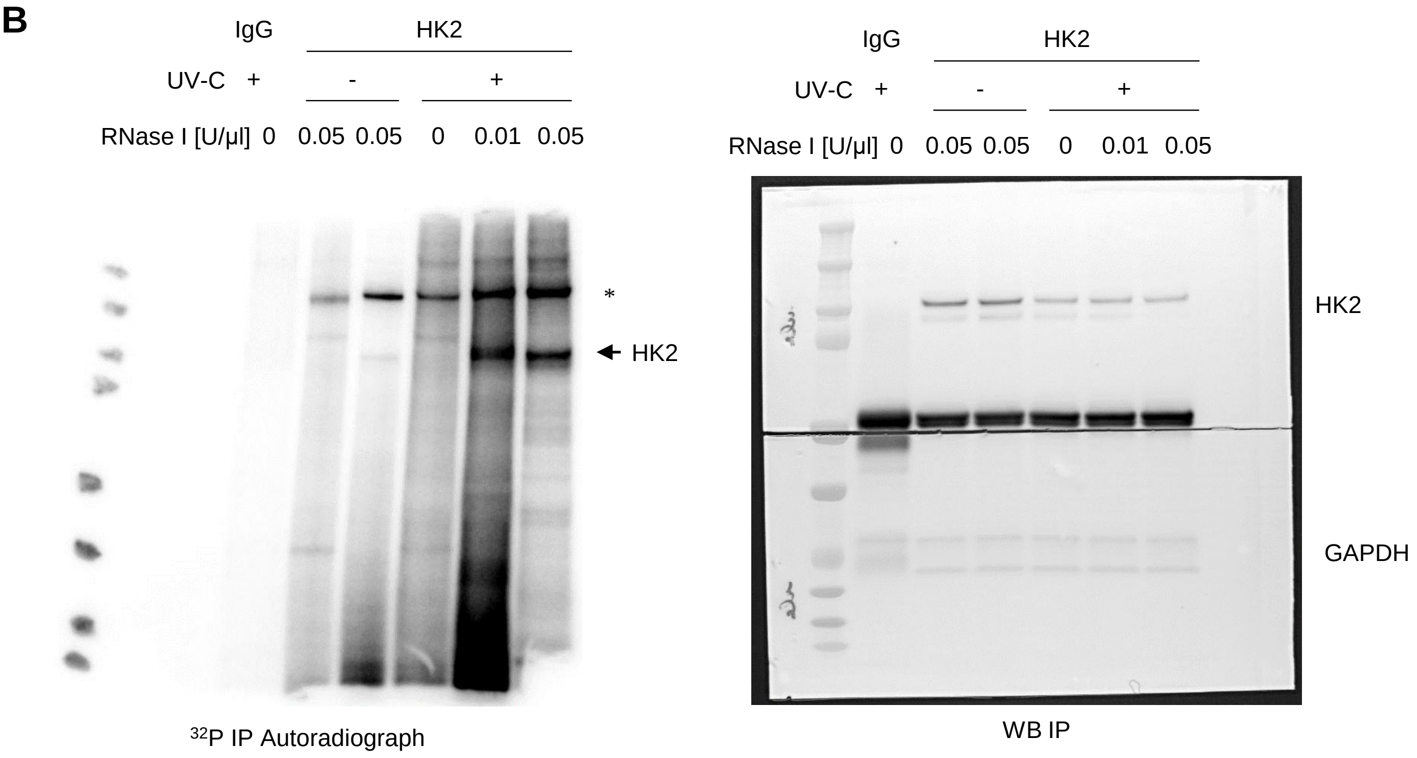
B
IgG
HK2
UV-C
+
-
+
RNase I [U/µl]
0
0.05
0.05
0
0.01
0.05
*
HK2
32P IP Autoradiograph
IgG
HK2
UV-C
+
-
+
RNase I [U/µl]
0
0.05
0.05
0
0.01
0.05
HK2
GAPDH
WB IP

Figure 3

C

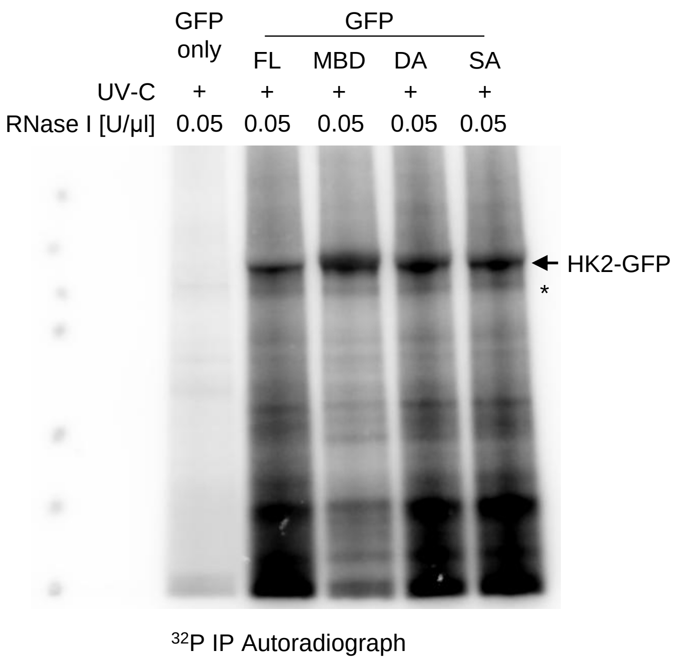

Figure S5E

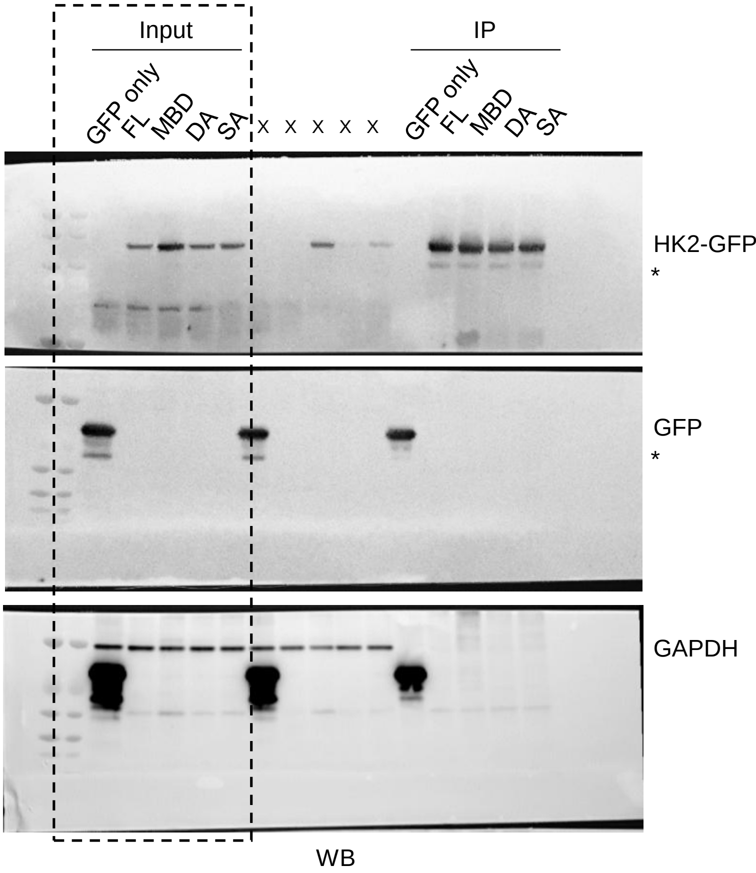

D

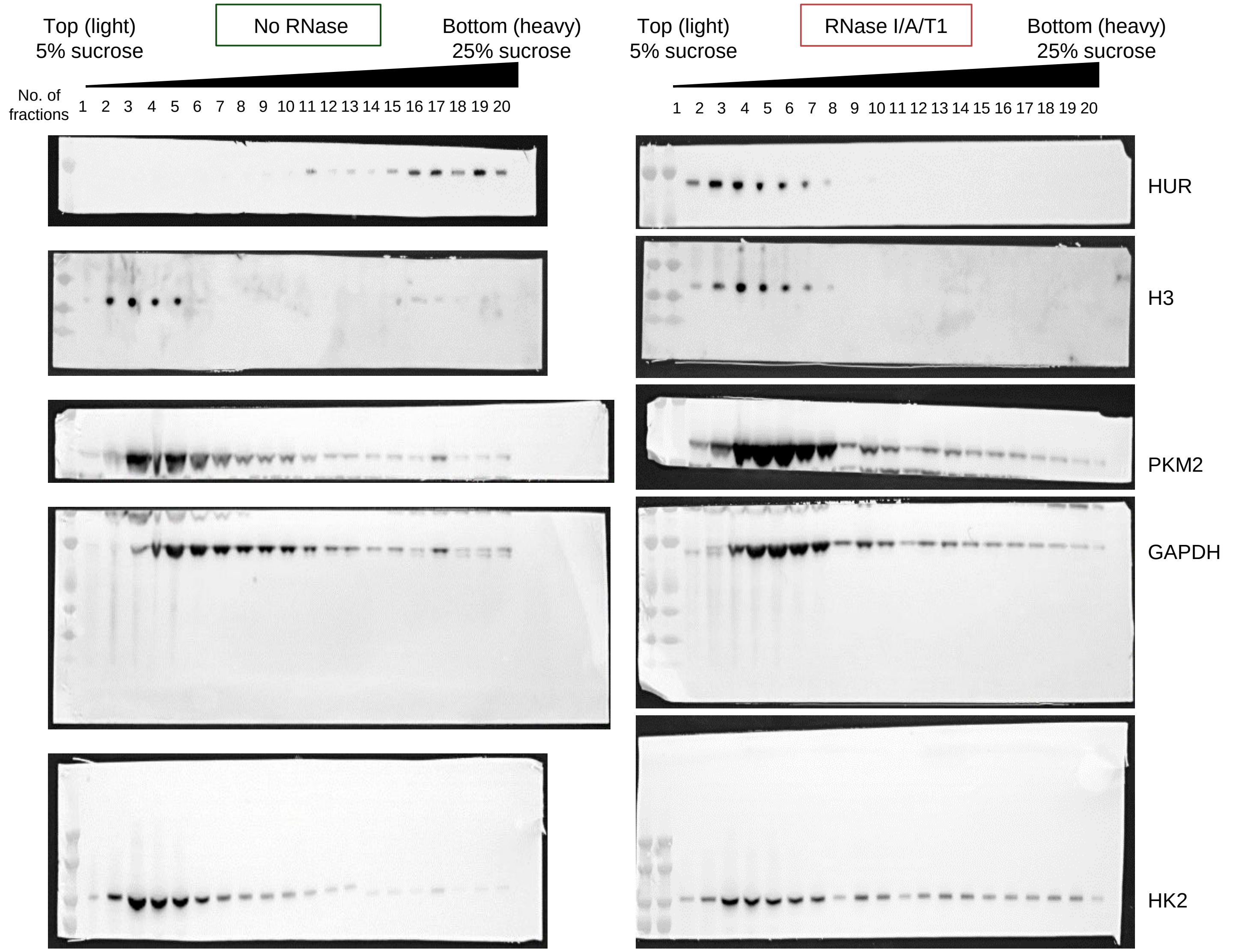

Figure 5

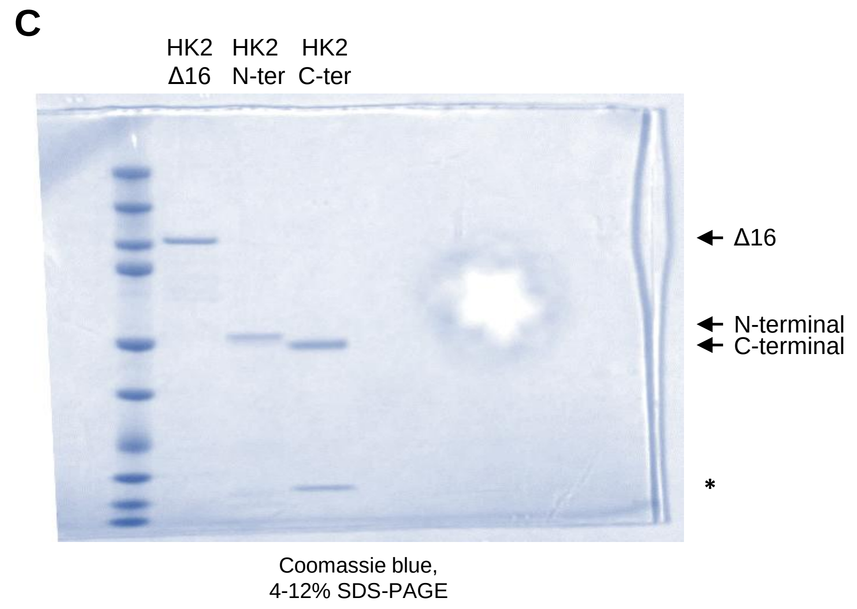
C
HK2 Δ16
HK2 N-ter
HK2 C-ter
Δ16
N-terminal
C-terminal
*
Coomassie blue,
4-12% SDS-PAGE

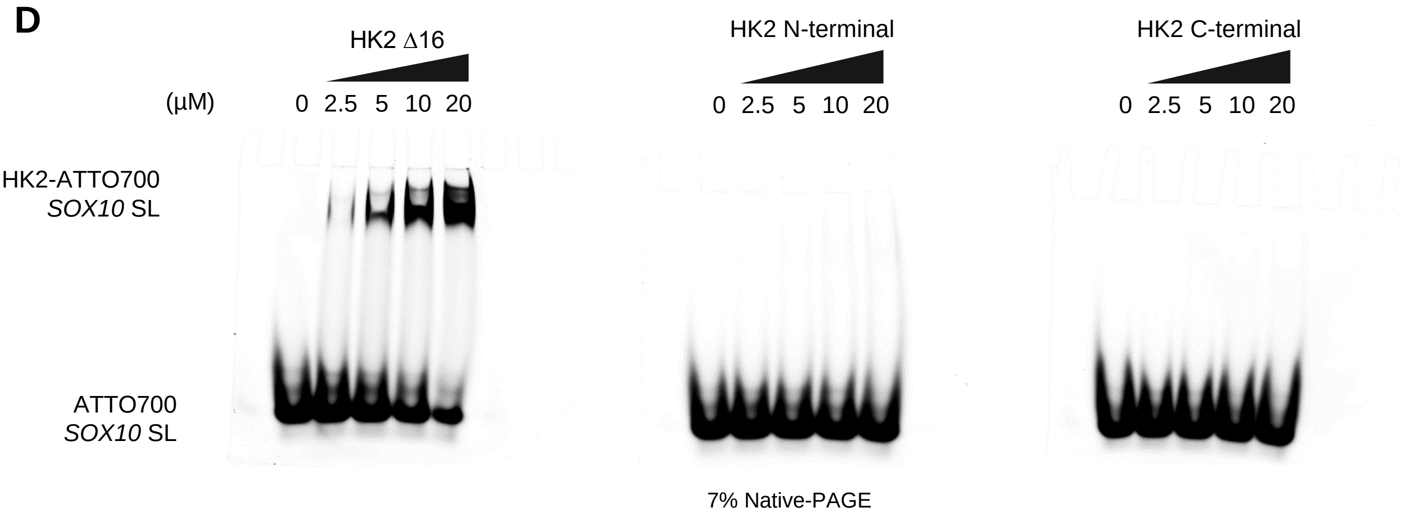
D
HK2 Δ16
HK2 N-terminal
HK2 C-terminal
(μM)
0 2.5 5 10 20
0 2.5 5 10 20
0 2.5 5 10 20
HK2-ATTO700
SOX10 SL
ATTO700
SOX10 SL
7% Native-PAGE

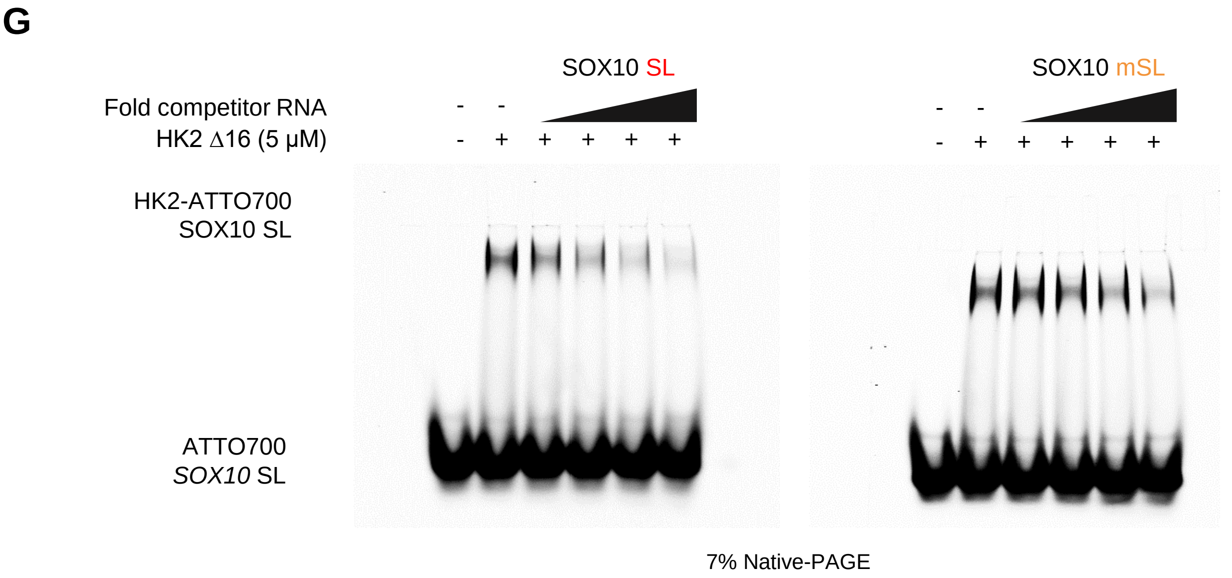
G
SOX10 SL
SOX10 mSL
Fold competitor RNA
HK2 Δ16 (5 μM)
- -
- + + + + +
- -
- + + + + +
HK2-ATTO700
SOX10 SL
ATTO700
SOX10 SL
7% Native-PAGE

Figure 6

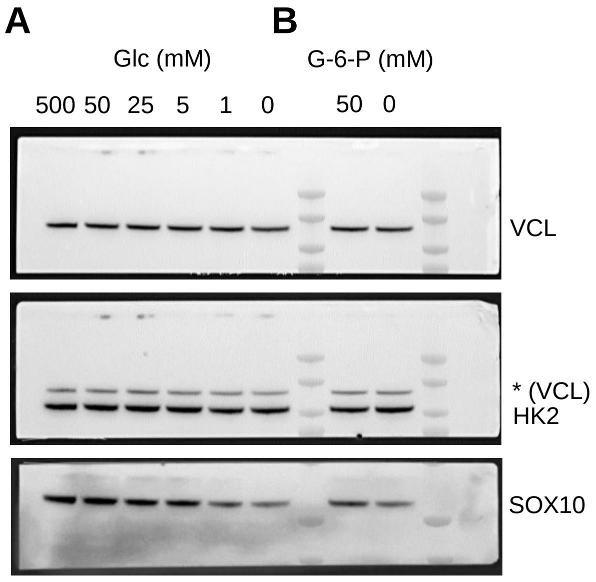

Figure 7

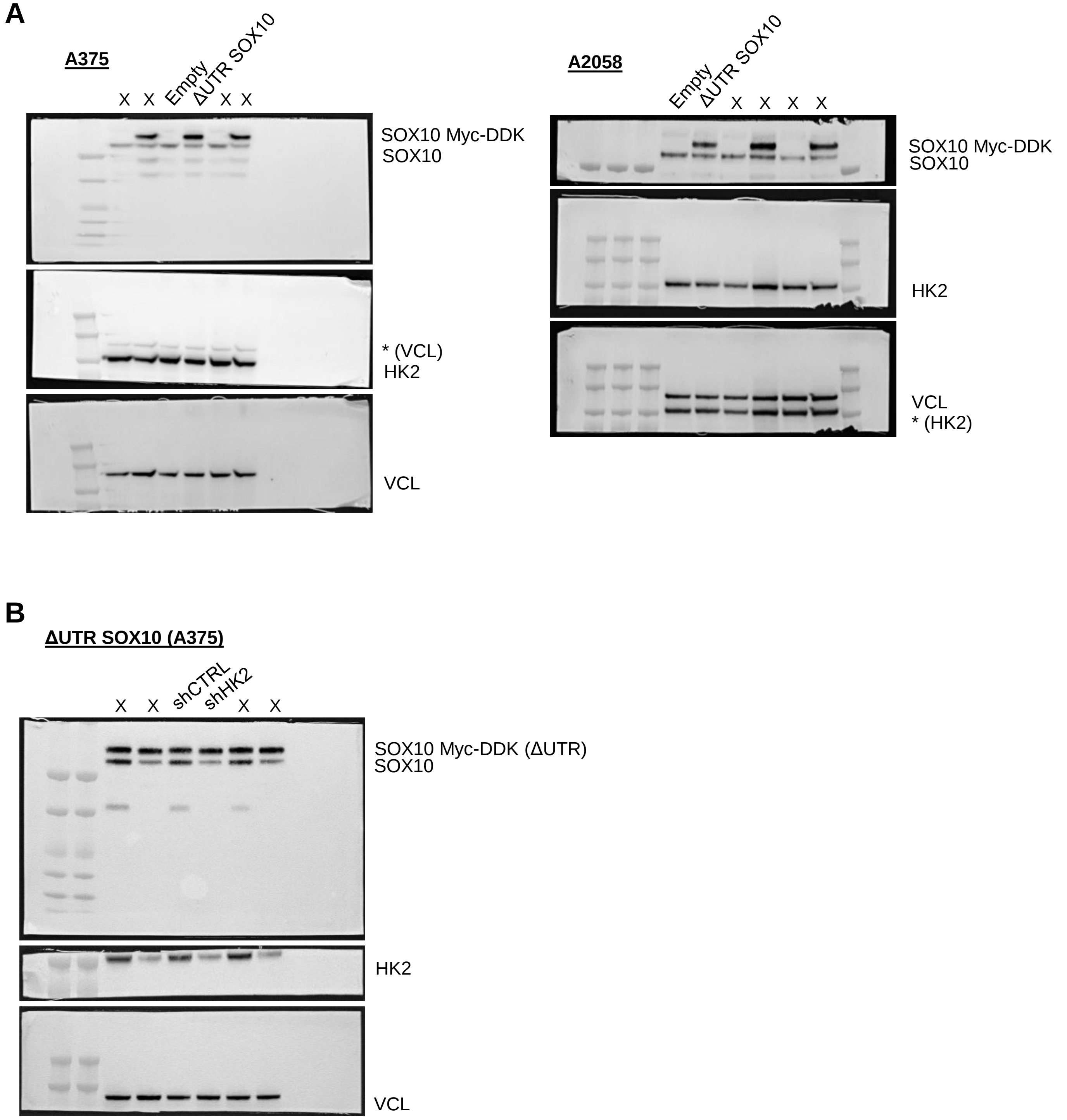
A
A375
X X Empty ΔUTR SOX10 X X
SOX10 Myc-DDK
SOX10
* (VCL)
HK2
VCL
A2058
Empty ΔUTR SOX10 X X X X
SOX10 Myc-DDK
SOX10
HK2
VCL
* (HK2)
B
ΔUTR SOX10 (A375)
X X shCTRL shHK2 X X
SOX10 Myc-DDK (ΔUTR)
SOX10
HK2
VCL

Supplementary

Figure S1

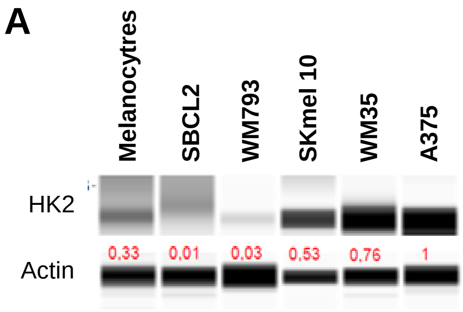
A
Melanocytres
SBCL2
WM793
SKmel 10
WM35
A375
HK2
0,33
0,01
0,03
0,53
0,76
1
Actin

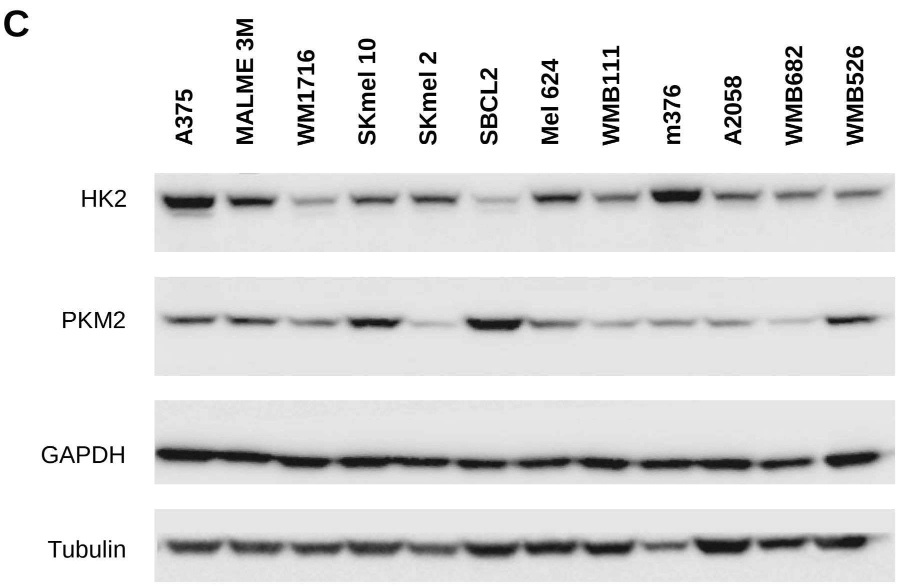
C
A375
MALME 3M
WM1716
SKmel 10
SKmel 2
SBCL2
Mel 624
WMB111
m376
A2058
WMB682
WMB526
HK2
PKM2
GAPDH
Tubulin

Figure S2

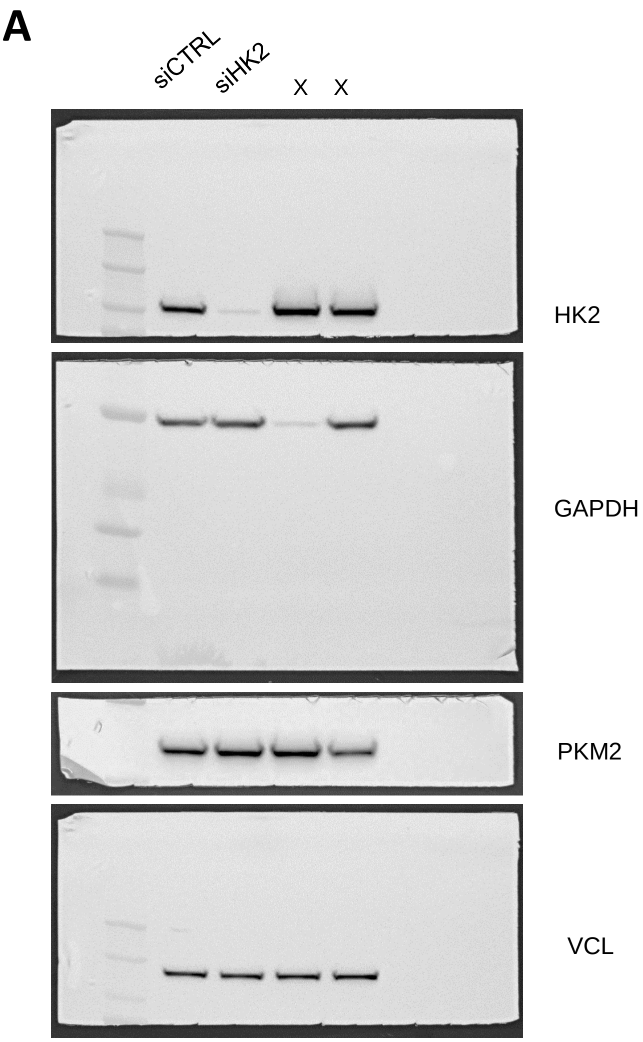

Figure S3

C

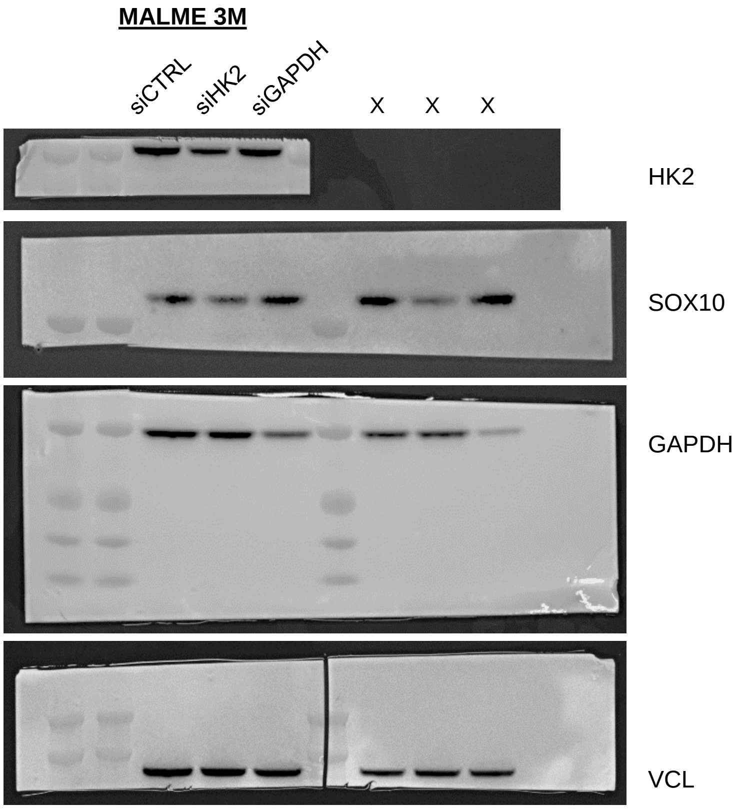

Figure S5

A

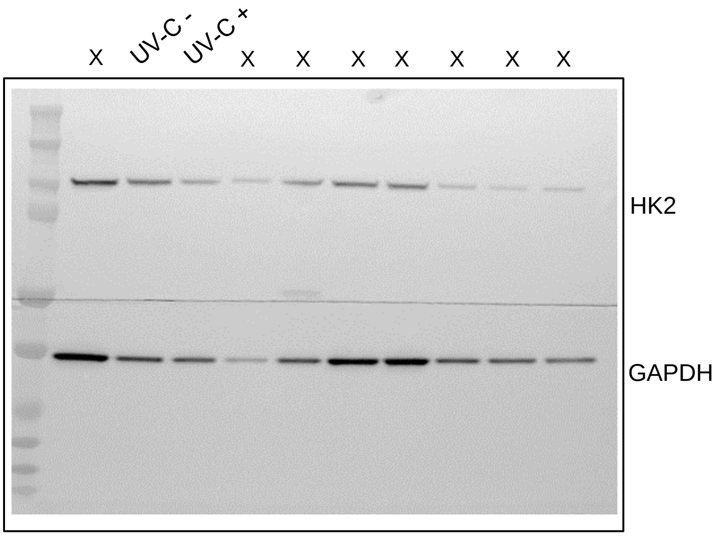

WB Input

Figure S5

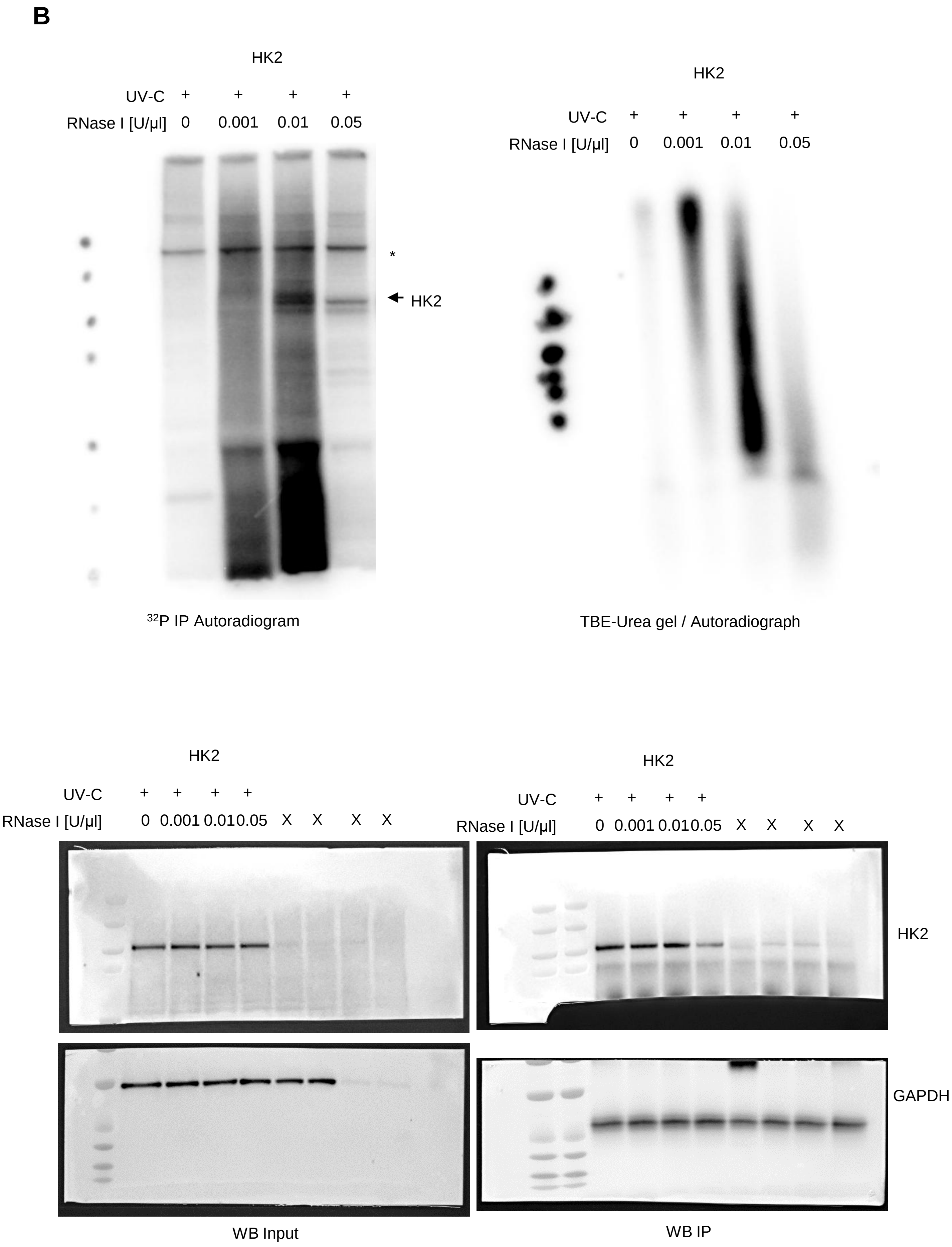

B
HK2
UV-C + + + +
RNase I [U/µl] 0 0.001 0.01 0.05
*
HK2
³²P IP Autoradiogram
HK2
UV-C + + + +
RNase I [U/µl] 0 0.001 0.01 0.05
TBE-Urea gel / Autoradiograph
HK2
UV-C + + + +
RNase I [U/µl] 0 0.001 0.01 0.05 X X X X
WB Input
HK2
UV-C + + + +
RNase I [U/µl] 0 0.001 0.01 0.05 X X X X
HK2
GAPDH
WB IP

Figure S5

C

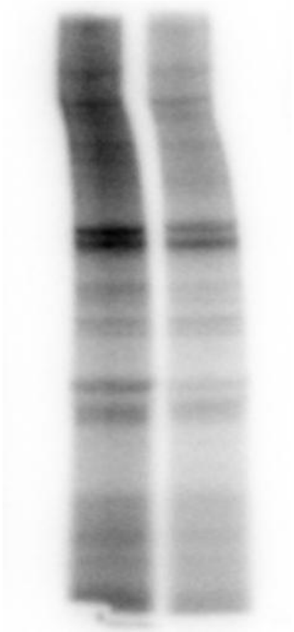

^{32}P IP Autoradiograph

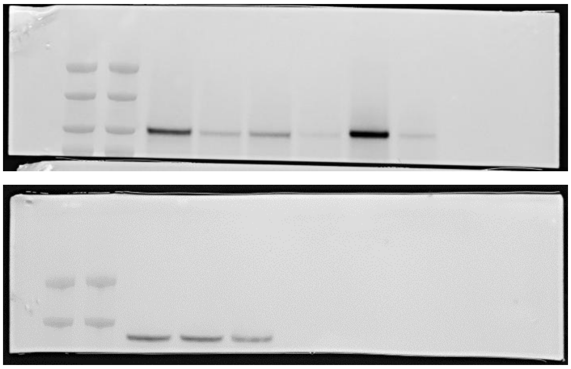

D

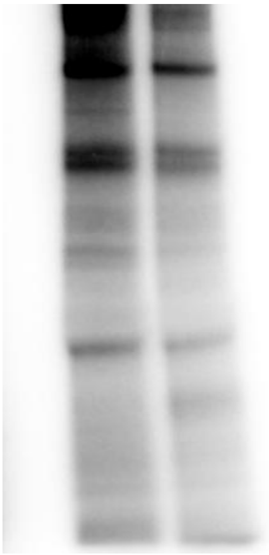

^{32}P IP Autoradiograph

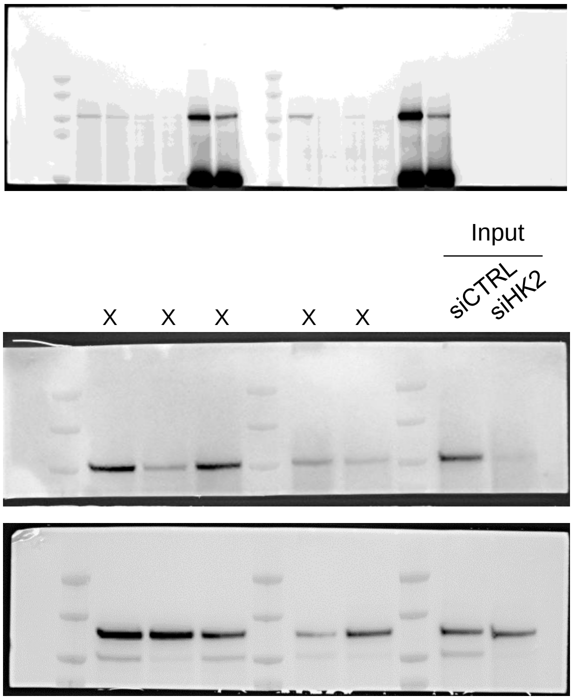

E

Figure S5E is presented together with Figure 3C (right panel), where indicated.

Figure S7

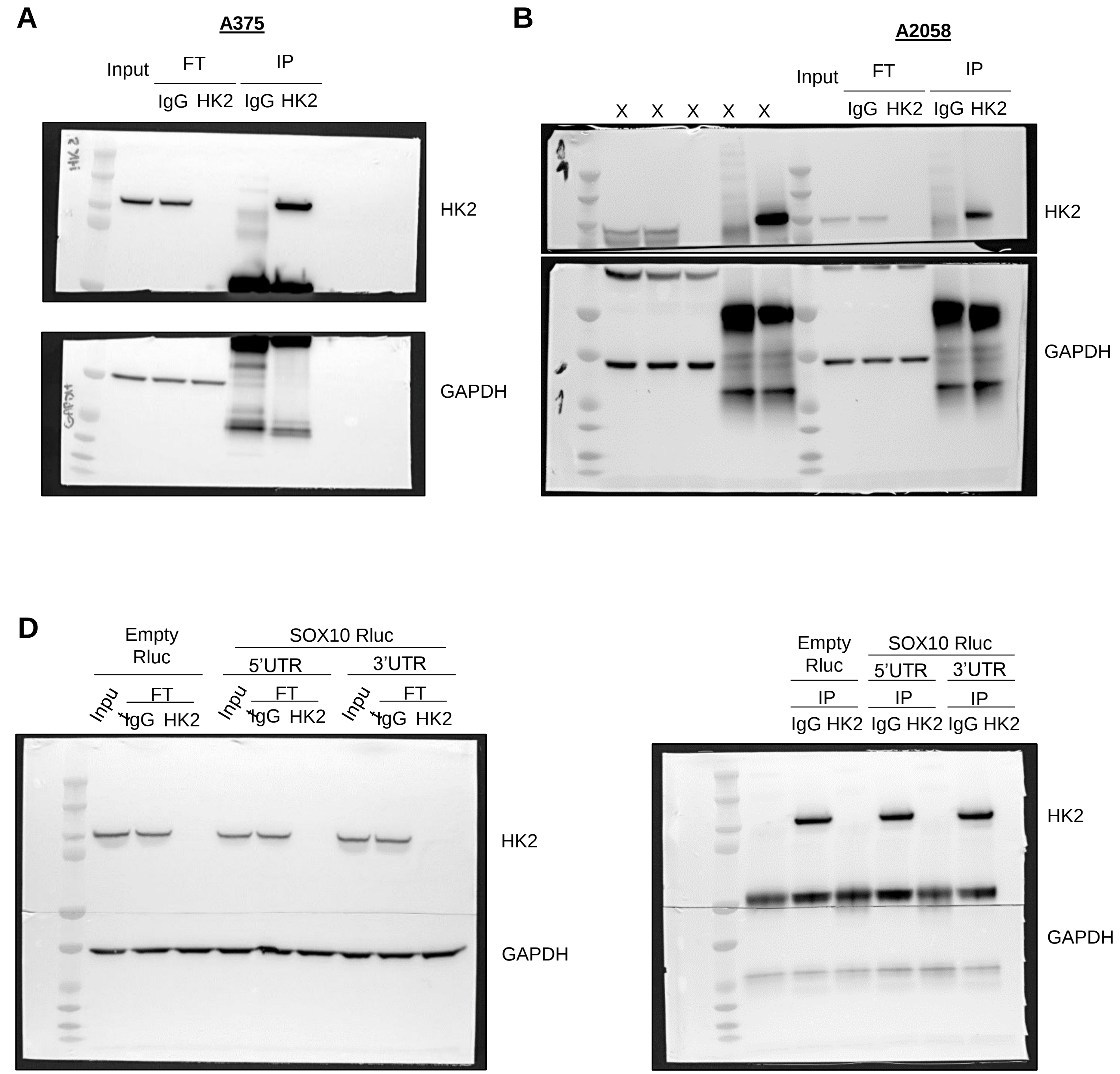
A
A375
Input
FT
IP
IgG HK2 IgG HK2
HK2
GAPDH
B
A2058
X X X X X
Input
FT
IP
IgG HK2 IgG HK2
HK2
GAPDH
D
Empty Rluc
SOX10 Rluc
5’UTR
3’UTR
Input
FT
IgG HK2
HK2
GAPDH
Empty Rluc
SOX10 Rluc
5’UTR
3’UTR
IP
IgG HK2 IgG HK2 IgG HK2
HK2
GAPDH

Figure S7

E

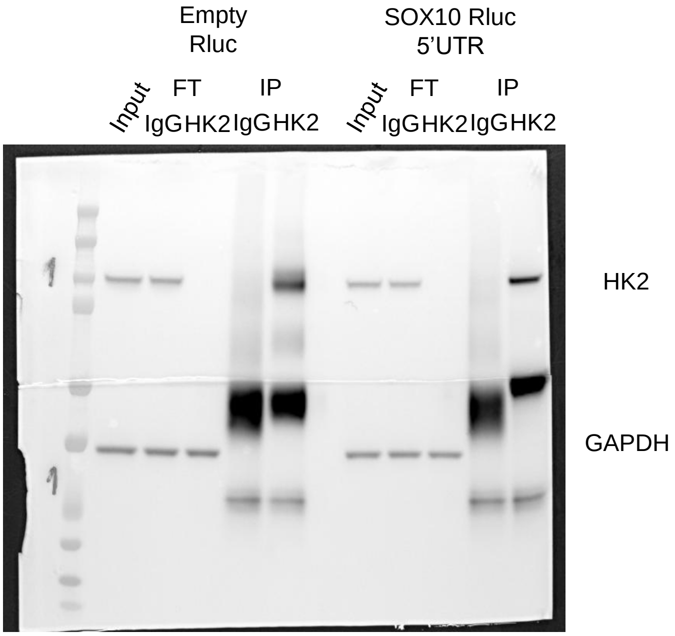

Empty Rluc
SOX10 Rluc 5'UTR
Input
FT
IgG
HK2
IP
IgG
HK2
Input
FT
IgG
HK2
IP
IgG
HK2
HK2
GAPDH

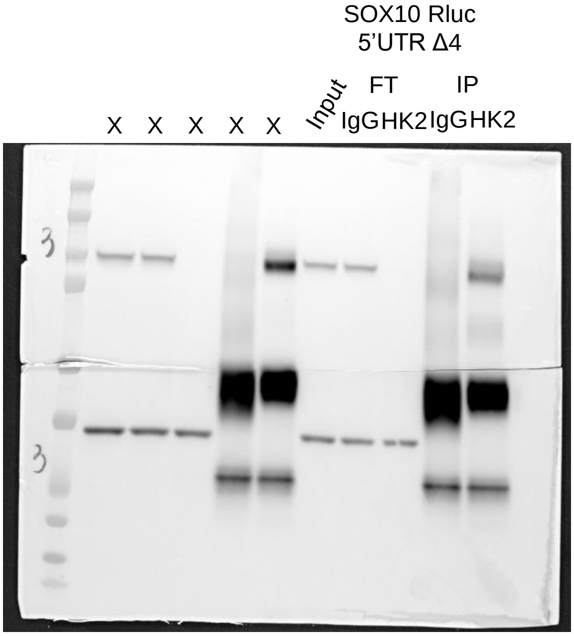

SOX10 Rluc 5'UTR Δ4
X X X X X
Input
FT
IgG
HK2
IP
IgG
HK2

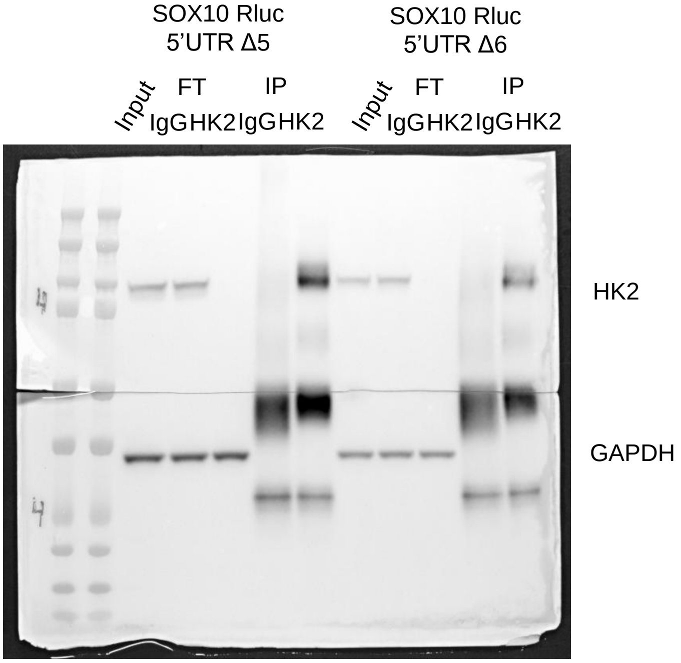

SOX10 Rluc 5'UTR Δ5
SOX10 Rluc 5'UTR Δ6
Input
FT
IgG
HK2
IP
IgG
HK2
Input
FT
IgG
HK2
IP
IgG
HK2
HK2
GAPDH
