# Supplementary material for: Hexokinase 2 is an RNA-binding protein that regulates mRNA translation independently of glycolysis and induces melanoma cell proliferation
Source: PLoS Biol. 2025 Sep 16;23(9):e3003364. doi: 10.1371/journal.pbio.3003364 (PMC12494293; doi:10.1371/journal.pbio.3003364)
Supplement: Attachment — Submitted filename: 149877_1_rebuttal_3193046_sm7sl4.pdf [file pbio.3003364.s031.pdf]

Revision Plan

Review COMMONS

Manuscript number: RC- 2024-02647
Corresponding author(s): Stéphan VAGNER

Reviewer's comments in black
Our responses in blue

1. General Statements

We would like to thank all three reviewers for their constructive suggestions. We were very excited to read that the reviewers considered the data well described and convincing and our findings representing an important information.
All new additions and changes to the manuscript are explained in our point-by-point response below.

Reviewer #1

(Evidence, reproducibility and clarity):
Summary:
In this work, Dian et al. experimentally validate the RNA-binding activity of hexokinase 2 (HK2) in melanoma cells, identify a HK2-binding site within the 5'UTR of the SOX10 mRNA, and demonstrate that HK2 positively regulates the translation of this mRNA. Finally, they provide evidence for the role of the HK2-SOX10 axis in melanoma cell proliferation and colony formation.
Major comments
I find the conclusions to be well substantiated by the data, and I don't believe additional experiments are necessary.

(Significance):
Over the last decade, hundreds of candidate RNA-binding proteins (RBPs) without identifiable RNA-binding domains or known RNA-related functions have been identified through high-throughput proteomics methods. Many of these non-canonical RBPs are enzymes involved in intermediary metabolism (PMID: 22658674, PMID: 26632259, PMID: 37045843). To date, the biological significance of their interaction with RNA remains unknown for most of these enzymes. In this work, the authors validate the interaction of the glycolytic enzyme HK2 with RNA and provide mechanistic insights into its role as a translational regulator, adding a valuable example to the growing list of enzymes with non-canonical roles in gene regulation. The data is particularly relevant for RNA biologists, especially those focusing on non-canonical RBPs or the regulation of protein synthesis.

Key areas of my expertise are: gene regulation and systems biology of RBPs.

Reviewer #2

(Evidence, reproducibility and clarity):

Summary:

In the present manuscript, Dian et al. identified a novel function of Hexokinase 2 (HK2). Beyond its established role in glycolysis during the initial step of glucose phosphorylation, the authors demonstrate that HK2 functions as a bona fide RNA-binding protein capable of regulating mRNA translation in human cancer cells. Notably, HK2 specifically binds to the 5' UTR of SOX10 mRNA, influencing cell proliferation.

(Significance):

The authors compellingly demonstrate that the metabolic enzyme HK2 can bind RNAs and regulate translation, revealing a moonlighting function and expanding our knowledge of non-canonical RNA-binding proteins. They employ a range of approaches to support the conclusion that HK2 interacts with RNA, providing solid and convincing data. However, a limitation of the study is that only one target has been shown to be regulated by HK2, and the link to HK2's metabolic function is missing, which could have provided a more comprehensive understanding of its dual roles.

The present manuscript provides new evidence that hexokinase 2 (HK2), traditionally known for its role in glycolysis, also functions as an RNA-binding protein. This finding introduces a conceptual advance in the field of post-transcriptional regulation and RNA-binding proteins, revealing novel functional dimensions of glycolytic enzymes.

My expertise encompasses RNA biology and post-transcriptional regulation in both prokaryotic and eukaryotic systems, with a particular focus on the role of RNA-binding proteins.

Reviewer #3

(Evidence, reproducibility and clarity):

Summary

The manuscript by Dian et al. reveals that the glycolytic enzyme hexokinase 2 (HK2) exhibits RNA-binding activity and regulates mRNA translation in melanoma. HK2 is enriched in polysome fractions. By using KD studies, the authors show changes in the translatome in melanoma cells upon loss of HK2. SOX10 was observed to be translationally downregulated in response to HK2 KD. While SOX10 mRNA levels were unchanged, reduced protein levels were observed. HK2 binding to SOX10 mRNA was confirmed using different assays, including 2C and CLIP. RIP qPCR was employed to validate HK2 binding to SOX10. Software-based prediction tools were used to analyze HK2-SOX2 interaction and the binding region was localized to the SOX10 5'-UTR. Further studies using reporter assays and deletion mutants were conducted, showing that the 3'-part of the SOX10 5'-UTR is most likely to be involved in binding to HK2. In the final part of the paper, the authors use CFU and proliferation assays to show that HK2-dependent translation regulation of SOX10 plays a role in regulating melanoma cell aggressiveness.

(Significance):

The authors of this study comprehensively report how the glycolytic enzyme HK2 binds to and regulates SOX10 mRNA. However, some of the data will need clarification and additional

experiments will be needed to fully support the authors' hypothesis. Potential caveats of the study should be discussed in greater detail.

2. Description of the planned revisions

Reviewer #1

Minor Comments:

1. Figures 2A and S3B: Error bars are missing.

This will be corrected.

5. Figure 4D:
Validation of SOX10 knockdown efficiency is missing. Could the relatively mild decrease in PLA signal in cells transfected with siSOX10 (Fig. 4D) be due to a mild knockdown?

The validation of SOX10 knockdown efficiency will be included in Figure 4.

Reviewer #2

Major comments:

- In the Western blot analysis of SOX10 protein (Figure 2) the authors should present the SOX10 levels without arbitrarily normalizing the control to 1: the variability of the control sample should be reported to ensure accurate interpretation of the data. The authors should confirm the effect of the KH2 knock-down on SOX10 expression, by using an alternative siRNA, to confirm the specificity of the regulation.

The data on Figure 2 will be presented without the arbitrarily normalizing the control to 1. Additionally, the effect of the KH2 KD on SOX10 expression had also been confirmed by the use of different siRNAs (A) and by the use of a short hairpin RNA (shRNA) targeting HK2 (shHK2) in A375 cells (B), which was compared to a control shRNA (shCTRL). Both results will be added to the manuscript and are presented below.

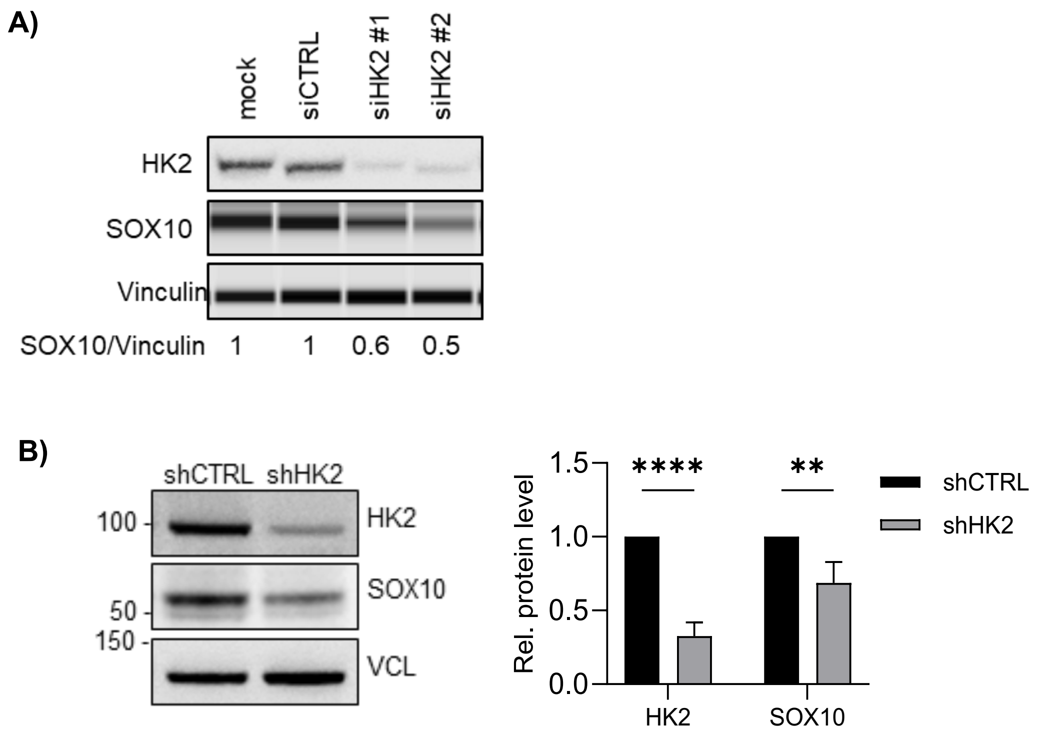

- Although the CLIP experiment (Figure 3B) using a high concentration of RNase I effectively determined the specificity of the protein-RNA complexes, could the authors provide insights into the potential origin of the non-specific band observed? The authors observe the same band of HK2 when using the lowest and the highest concentration of RNase I, the authors should perform more dilutions of RNase I to clearly detect a smear reduction and HK2 band sharpening. In Figure 1C, controls with IgG and no RNase I (at least for the WT HK2-GFP) should be provided; alternatively to the IgG, a control with samples expressing only GFP (not fused to HK2) should be used.

Regarding Figure 3B, HK2 depletion by targeted siRNA will be performed to demonstrate the specificity of the band. More RNase I dilutions will also be included to clearly detect the smear. However, it's possible to see the band sharpening when increasing the concentration of RNase I. Information relative to the RNase I concentration had been included in the methods session. This information has also been included in the figure.
Regarding Figure 3C (not 1C), CLIP experiments will be performed using GFP only as control for the HK2-GFP bands.

- The authors should clarify the selection criteria for the 84 cancer-relevant mRNAs and detail their overlap with HK2 targets. Additionally, regarding the target validation in Figure S3B, the authors should provide information on the number of replicates and the statistical analysis used. Was SOX10 mRNA selected because, unlike the other targets tested, no difference was observed at the total RNA level (Figure S3B)? Additional information should be provided on the selection of

SOX10, especially since it was not identified as significantly regulated in the translatome analysis shown in Figure 1A.

RT2 Profiler PCR Arrays were used to monitor the expression of 84 relevant, pathway-focused genes. The RT² Profiler™ PCR Array Human Epithelial to Mesenchymal Transition (EMT) was selected due to the variety of cancer-relevant genes. The target list includes genes involved in cell differentiation and development, cell growth and proliferation, cell migration, transcription factors, among others. This list can be found at: https://geneglobe.qiagen.com/us/product-groups/rt2-profiler-pcr-arrays/PAHS-090Z.

We did not observe an overlap between the 84 mRNAs analyzed by qPCR and the 59 identified targets. Among the mRNAs that were specifically translationally regulated upon siRNA-mediated HK2 depletion, we were particularly interested in the SOX10 mRNA. This mRNA encodes a well-known transcription factor that plays an important role in the differentiation of neural crest precursors to the melanocytic lineage (Simões-Costa et al, 2015). However, SOX10 has also been shown to be involved in the regulation of tumor initiation (Huang et al, 2015; Blokzijl et al, 2016; Gambichler et al, 2016; Huang et al, 2015), maintenance and melanoma cell proliferation (Capparelli et al, 2022; Cronin et al, 2013; Shakhova et al, 2012). Additionally, this transcription factor has been identified as a mediator of phenotype switching of melanoma cells, as well as a factor of resistance to immunologic cell death (Rosenbaum et al, 2024) and drug resistance against current targeted therapies (BRAF and MEK inhibitors) (Glasheen et al, 2023; Capparelli et al, 2022; Han et al, 2018; Sun et al, 2014). Thus, this information has been included in the manuscript to further clarify the selection of the SOX10 mRNA for further validation. Additionally, the target validation in Figure S3B has been performed once. Replicates will be performed and included in the figure.

- Discussion: The authors could further discuss the potential mechanisms by which HK2 interacts with RNA, comparing it to other metabolic proteins that are known to bind RNA and explore how it can promote mRNA translation.

Previous RNA interactome studies revealed that many of the newly identified RBPs do not harbor well-defined RBDs. Indeed, numerous metabolic enzymes were found to bind RNA in the vicinity of their substrate-binding pockets, as mapped by RBDmap and in accordance with previous observations (Castello et al., 2016; Nagy and Rigby, 1995). For example, Rossmann fold, a di-nucleotide domain typically associated with the binding of nucleotides such as the nicotinamide adenine dinucleotide (NAD) (Rossman et al., 1975), has been shown to mediate binding to RNA (particularly in dehydrogenases, such as GAPDH) (Nagy and Rigby, 1995). However, many other domains remain to be validated regarding their ability to direct enzyme binding to RNA. Accordingly, HK2 does not harbor any recognizable RNA-binding domain. However, we demonstrated in this manuscript that HK2 directly binds RNA in living cells and, with the use of HK2 mutants, we also demonstrated that this RNA-binding activity does not rely on the glucose-binding domain, on the enzyme's kinase activity, or on its localization at the outer mitochondrial membrane (OMM).

Metabolic enzymes, particularly glycolytic enzymes, have been described to bind to a wide variety of RNA types (e.g., mRNAs, tRNAs, vRNAs), sequences (e.g., AU-rich sequences), regions

(5'UTR, 3'UTR, CDS) and secondary structures (e.g., stem loop), holding crucial functions in regulating co- and post-transcriptional steps of gene expression by acting as trans-acting factors to control the RNA fate (Wegener and Dietz, 2022). Indeed, a few glycolytic enzymes have been found to regulate mRNA translation either by direct binding to RNAs (including tRNAs (Sy et al., 2018; Baleva et al., 2017; Baleva et al., 2015; Entelis et al., 2006; Singh and Green, 1993)) and/or by interacting with ribosomes (Kejiou et al., 2023; Simsek et al., 2017). In this manuscript, we found that HK2 binds and affects SOX10 mRNA translation in a 5'UTR-dependent manner. Whether this interaction is sequence- or structure-dependent remains to be defined. Given the importance of 5'UTRs for translation initiation, it is conceivable that HK2 may facilitate SOX10 mRNA translation initiation by promoting ribosome association with the mRNA. Accordingly, we do observe that HK2 depletion decreases SOX10 mRNA association with translationally active polysomes in melanoma cells. However, further studies are required to test whether HK2 facilitates ribosomal assembly, for example, through the recruitment of eIF4F factors to the SOX10 5'UTR. This discussion will be added to the manuscript.

Reviewer #3

<u>Major Comments</u>

- As mentioned by the authors, SOX10 was not identified in the sequencing approach. It is important to know if the sequencing and qPCR data are in line. What is the overlap of the 59 identified targets and the 84 mRNAs analyzed by qPCR? Unfortunately, the sequencing data is somewhat unconnected to the rest of the manuscript. While it is of potential interest, the 59 hits do not play a role in the follow-up experiments. Not even validation of promising targets was performed. In contrast, Sox10 is analyzed and followed-up on, a hit that was not identified in the sequencing approach. The authors need to better connect these parts and outline potential caveats and limitations of the sequencing data. Why was it not further investigated or used? At the moment the sequencing data does not add much to the take home messages of the manuscript.

We did not observe an overlap between the 59 identified targets and the 84 mRNAs analyzed by qPCR. The sequencing approach allowed the identification that HK2 regulates mRNA translation independently of other GEs or potential disturbances in the glycolysis due to the enzyme's KD. Indeed, one can appreciate that analysis of mRNAs recruited to heavy polysome fractions in GE-depleted cells under normoglucidic conditions allowed the identification that the subsets of differentially translated mRNAs are different following GAPDH, PKM2 and HK2 depletion. Additionally, analysis of mRNAs recruited to heavy polysome fractions in cells under glycolysis inhibition (galactose) and HK2-depleted cells under normoglucidic conditions demonstrated that the subsets of differentially translated mRNAs are different following glycolysis inhibition and HK2 depletion. In fact, only ~20% of mRNAs deregulated upon HK2 KD are also deregulated in galactose. Together, these results indicate that HK2 not only regulates mRNA translation, but also that the translation regulation by HK2 is partly independent of the metabolic status of the cells. We did not proceed with the validation of the translationally deregulated mRNAs found in the sequencing approach because they are not currently recognized as cancer-relevant. However, promising targets can be validated. Given the scope of our study, we focused on mRNAs with established roles in cancer. Therefore, by prioritizing the mRNAs already implicated in cancer pathways, we could focus on the identification of molecular mechanisms relevant to tumorigenesis and therapeutic intervention. For that reason, we performed, in parallel to the

sequencing approach, a small-scale targeted screening (qPCR array) approach to identify cancer-relevant mRNAs translationally regulated by HK2 that could have been missed in the large-scale sequencing approach. Indeed, SOX10 might have been identified in the small screening but not in a large-scale sequencing approach due to differences in sensitivity, specificity, and detection thresholds between the methods. In the qPCR approach, specific targets can be investigated, thus the assay is optimized to detect those particular genes. In contrast, the large-scale sequencing is designed to capture a broader range of transcripts. In this context, the data are typically processed to reduce false positives and remove background noise, which reduces the sensitivity of the technique for the detection of some true positive deregulated transcripts.

- While 2C and CLIP analysis has been thoroughly done, it would be great to add additional controls. Please conduct the experiments using HK2 KD cells to prove the specificity of the bands. In addition, for CLIP analysis more RNase concentrations (at least 3) should be used to see better collapsing of the band. I would suggest using a third condition with even less RNase. It is also not clear how much RNase was used. Please add this information to the figure or figure legend.

HK2 depletion by targeted siRNA will be performed to demonstrate the specificity of the band. More RNase I dilutions will also be included to clearly detect the smear. However, it's possible to see the band sharpening when increasing the concentration of RNase I. Information relative to the RNase I concentration had been included in the methods session. This information has also been included in the figure.

- An additional control is missing in Figure 3C. Please also conduct the experiment using an empty vector control to prove the specificity of the detected HK2-GFP band. Especially considering the unspecific band detected closely to the GFP-HK2 band.

CLIP experiments will be performed using GFP only as control for the HK2-GFP bands.

- The authors should make use of a second cell line for their key experiments conducted in Figure 3 and 5. This would make the conclusions of the manuscript much stronger. Especially as a second cell line has been used to generate data of Figure 4. In principle, it would also be of interest to see if this mechanism is melanoma-specific. Is this mechanism also active in healthy melanocytes or is it specific to invasive melanoma cells? The authors should at least test HK2-Sox10 interaction by RIP-qPCR and general RNA-binding activity of HK2 (2C, CLIP) in healthy melanocytes.

Key experiments will be performed in A2058 melanoma cell line, as suggested.
The Human Protein Atlas (proteinatlas.org) states that HK2 is low expressed in healthy melanocytes. Accordingly, HK2 is described to be mainly expressed in adipose tissue, skeletal, and cardiac muscles (Wilson, 2003). Additionally, this enzyme is frequently found upregulated in cancer cells (Wang et al, 2017; Kudryavtseva et al, 2016; Patra et al, 2013; Liu et al, 2013; Wolf et al, 2011). Altogether, these data suggest that the mechanism found in this manuscript is not active in healthy melanocytes, but in melanoma cells.

- As the authors nicely identified a 100 bp window within the 5'UTR of SOX10 that is binding to

HK2, they should conduct an EMSA assay to further validate and strengthen these results using recombinant HK and an RNA oligo.

Recombinant HK2 will be purified and for the execution of EMSA experiments.

- While the authors take great care to exclude metabolism-related effects while analyzing translatome data (by using galactose and different KD conditions) it must be stated that KD of HK2 will impact the overall cellular transcriptome and translatome by affecting cellular metabolism. Thus, the changes observed might very well be secondary. This problem should at least be discussed in the manuscript. I appreciate that experimentally addressing this problem is rather challenging. For a start, the changes in metabolism upon HK2 KD should be analyzed by Seahorse, as done for galactose/glucose culturing conditions.

The seahorse experiment performed included treatment with 2DG, an HK2 inhibitor. HK2 inhibition with 2DG decreases ECAR (which is a measure of lactic acid - lactate production - levels). Instead, lactate assays will be performed in the context of HK2 KD. Additionally, the impact of HK2 KD in the overall cellular transcriptome and translatome by affecting cellular metabolism will be further discussed in the manuscript.

- Could the authors perform further analysis on the 59 targets that are specifically identified as being regulated by HK2. Are these targets from the same or related pathways? Are there sequence or structural similarities?

Pathway enrichment analysis, along with sequence motif and RNA secondary structure (RSS) motifs analyses will be performed for the 59 mRNA targets that are specifically identified as being regulated by HK2.

- Sox10 overexpression does compensate for loss of HK2 as can be seen in Figure 6B-C. However, this effect could be secondary and not directly related to the translational reductions observed. SOX10 is a very strong TF and overexpression will lead to very unphysiological protein levels. Can the authors comment on this? Especially considering the proliferation data shown for A2058. It can be observed that overexpression of Sox10 alone already has major effects on the cellular behavior.

SOX10 is a strong transcription factor, and its overexpression could indeed lead to unphysiological protein levels that could potentially cause secondary effects unrelated to the specific phenotype under investigation. However, although SOX10 overexpression alone has an effect in the proliferation in A2058 cells, these effects are consistent with SOX10's known role as a critical regulator of melanoma cell proliferation (Capparelli et al, 2022; Cronin et al, 2013; Shakhova et al, 2012). These observations will be included in the discussion session.

- While the functional assays performed are very interesting, the conclusions that can be drawn from them at the moment are minor. Additional functional experiments need to be conducted to conclude that oncogenic functions of HK2 are dependent on Sox10 translation (e.g. cell migration assays, in vivo assays). Alternatively, the statements need to be toned done.

*Additional functional experiments have been conducted for further investigation. Using 2D IncuCyte scratch-wound healing assays, we conducted migration assays in A375 cells upon HK2 KD for 72 hours (A). The cells and gap area were photographed every 12 hours, and the Relative Wound Density (%) measured. This assay was simultaneously performed in A375 cells that ectopically expressed SOX10, which were generated through the transduction of lentivirus particles carrying the SOX10 CDS (therefore without the sequences of the 5' and 3'UTRs). We observed that cells upon HK2 KD travelled significantly less than controls. However, ectopic expression of SOX10 did not reduce the effect observed upon HK2 KD. Conversely, depletion of HK2 in SOX10 transduced cells showed an even further decrease in the migratory capacity of the cells when compared to the control. p-values were calculated by ordinary two-way ANOVA with Turkey's multiple comparison test (SD, n = 3 biological replicates), and only significant differences within the same cell line are shown (*p ≤ 0.05; ** p ≤ 0.01).*

Our results are in accordance with different reports demonstrating that SOX10 expression inversely correlates with cell migration (Capparelli et al., 2022; Wouters et al., 2020). Besides, while SOX10 KD has been shown to promote cell migration, it has also been demonstrated to decrease melanoma cell proliferation and clonogenicity (Han et al., 2018; Sun et al., 2014). Indeed, in contrast to our findings from migration assays, we observed that ectopically expression of SOX10 in HK2 depleted cells rescues the ability of the cells to proliferate. Together, our results suggest that the effect of HK2 depletion on migration properties of melanoma cells is independent of the SOX10 UTR, while the effect of HK2 depletion on proliferation properties of melanoma cells requires the SOX10 UTR. Of note, HK2 KD only impacted the protein expression of endogenous SOX10, one additional evidence that HK2 affects SOX10 mRNA translation in a 5'UTR-dependent manner (n=3) (B). Figures A and B will be included as supplementary in the manuscript, and they can be found below.

A)

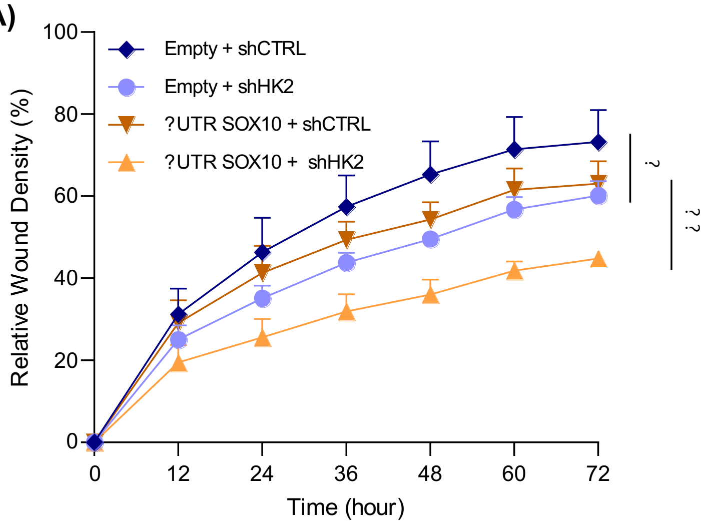

B)

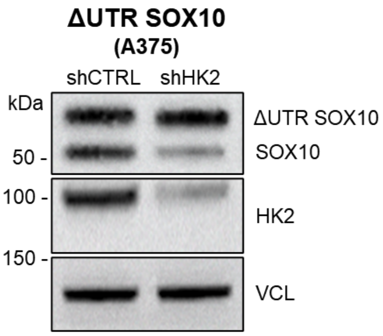

- While the authors thoroughly investigate the effects of HK2-Sox10 interaction on Sox10 translation, it would be of interest to briefly check the effects on HK2. Is enzyme activity impaired upon binding of Sox10?

Revision Plan

Indeed, RNA has been described to mediate the inhibition of the enzymatic activity of the glycolytic enzyme ENO1 in the context of stem cell differentiation (Huppertz et al, 2022). Undeniably, riboregulation may represent a widespread mechanism of biological control and should be investigated in the context of enzymes with RNA-binding activity. Although the effects of HK2-SOX10 mRNA interaction on the enzymatic activity of HK2 could briefly be investigated (e.g. in vitro), a potential riboregulation of HK2 and its biological significance requires for a more detailed exploration. We will, however, add a comment in the discussion section.

<u>Minor Comments</u>

- The authors conclude in the abstract that melanoma cells enhance glycolysis potentially because of the non-metabolic functions of HK2. This is a very strong statement considering the few CFU and proliferation data that is being shown. In addition, this statement is not sufficiently discussed in the discussion part. It is important to further discuss this part or at least tone down this statement.

This statement will be revised and further discussed in the manuscript.

- Figure 1B: after how many hours were the cells analyzed? What is the number of biological replicates for this experiment (should be >= 3). Please also provide a quantification of the western blot, including SD and statistical analysis.

Cells were harvested 48h after siRNA transfection. The experiment had been performed in triplicates, and statistical analyses for western blot quantifications will be included in the figure.

- For figure S1B, the authors provide a p-value in the text. Please also show a figure panel for the quantification, and not only representative IHC images. In addition, it is not clear what "low" and "high" are standing for in this panel. Presumably it is the melanoma stage. Please clarify.

In the panel S1B, "low" and "high" stand for HK2 expression. This information has been clarified in the figure legend, and the IHC quantification will be included.

- Figure S3A and S3B: the number of biological replicates is not clear. It should be at least >=3 and the SD should be shown.

The experiment had been performed only once. This information has been added to the figure and figure legend. Replicates will be performed and included in the figure.

- Figure 2A: the number of biological replicates is not clear. It should be at least >=3 and the SD should be shown.

This information has been updated in the figure legend. Further analysis of mRNA translation will be performed.

- Figure 6A: WB data should be quantified and depicted.

Western blot quantification will be included in the figure.

3. Description of the revisions that have already been incorporated in the transferred manuscript

Reviewer #1

Minor comments.

2. Page 8, Line 10:
"Strikingly, all mutants retained their ability to bind RNA, indicating that the interaction of HK2 with RNA is independent of its enzymatic activity, its ability to bind glucose, or its subcellular localization."
The statement that RNA binding is independent of HK2's subcellular localization seems too general. A more accurate description of the result would be: "RNA binding of HK2 does not rely on its association with the outer mitochondrial membrane."

The statement has been updated in the manuscript as follows:

"Strikingly, all mutants retained their ability to bind RNA, indicating that the RNA-binding activity of HK2 does not rely on its enzymatic activity, its ability to bind to glucose or its association with the OMM."

3. Page 8, Line 24:
"We next determined whether HK2 could specifically associate with SOX10 mRNA, which was identified to be translationally regulated by HK2."
I suggest revising this to: "... translationally regulated in HK2-depleted cells."

The statement has been revised as suggested.

4. Page 8, Line 30:
"Indicating that HK2 specifically binds to the SOX10 mRNA (Figure 4A and S7A)." Figure S7A does not appear to correspond to the mentioned result.

One additional figure was included as supplementary. This citation is now correct and corresponds to the mentioned results.

Reviewer #2

Major Comments

- Figure 1B: evaluate the possibility to move this panel in Supplementary Figures.

This panel has been moved to supplementary.

- In Figure 1C and 1D, the authors should clarify if the analysis of the translatomes the data are normalized on the level of expression of the corresponding RNA.

The translatome data were normalized to the level of expression of the corresponding RNA. This information has been included in the manuscript.

- The association of HK2 with polysomes appears to be predominant in the early fractions, including 80S monoribosome and light polysomes, but is absent in the heavier polysome fractions (unlike GAPDH and, to some extent, PKM2). Could this pattern be explained by a potential role for HK2 in translation initiation? It would be helpful if the authors could elaborate on these differences and provide further explanation. The authors should use EDTA to dissociate the polysomes and monitor the distribution of HK2 in this condition to verify the specificity of HK2 association with polysomes.

Although the mechanism by which GAPDH regulates mRNA translation is not fully elucidated, this glycolytic enzyme has been frequently described to impair translation through its binding to AU-rich sequences (AREs) within the 3'UTR of mRNAs (Galván-Peña et al, Nat Commun., 2019; Millet et al, J Immunol., 2016; Xu et al, J Clin Invest., 2016; Chang et al, Cell, 2013; Backlund et al, NAR, 2009; Yi et al, J Virol, 2000). However, GAPDH has also been shown to directly bind tRNAs, thus suggesting a potential involvement of this enzyme in translation elongation (Singh & Green, Science, 1993).
PKM, on the other hand, has been demonstrated to both stall and promote translation elongation of ribosomes on a subset of mRNAs (Kejiou et al, NAR, 2023, Simsek et al, Cell, 2017). Regarding translation stalling, PKM is proposed to act in cis (i.e. through its bound ribosome) and/or in trans (i.e. neighboring ribosomes). However, this process is proposed to happen by the direct association of the enzyme with the A-site of ribosomes, since PKM was shown to specifically interact with elongating ribosomes in the proximity of the A-site (Simsek et al, Cell, 2017). Indeed, translation elongation could be stalled by inhibition of the entry of tRNAs to the A site.
In this manuscript, we found that HK2 binds and affects SOX10 mRNA translation in a 5'UTR-dependent manner. Given the importance of 5'UTR sequences for translation initiation, it is conceivable that HK2 may facilitate SOX10 mRNA translation initiation by promoting/facilitating ribosome association with the mRNA. Accordingly, we do observe that HK2 depletion decreases SOX10 mRNA association with translationally active polysomes in melanoma cells. However, further studies are required to test whether HK2 facilitates ribosomal assembly, for example, through the recruitment of eIF4F factors to the SOX10 5'UTR.
We will add these different comments in the discussion. Also, the EDTA experiments had been performed and will has been included in the manuscript to demonstrate the specificity of HK2 association with polysomes. This experiment can be found bellow:

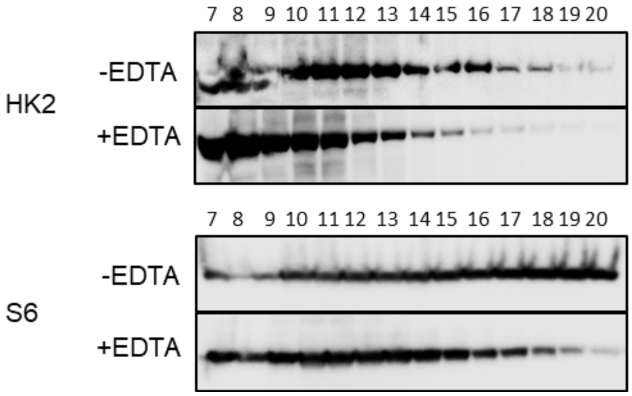

- In the RNA-immunoprecipitation experiments (Figure 4A), comparisons should not be made between different targets, but rather between the IgG and HK2 conditions within the same target. Alternatively, the efficiency of the primers should be validated to ensure consistency.

The efficiency of the primers was validated, and the Ct value of each RIP RNA fractions had been adjusted to the efficiency of the primers to ensure consistency. This information has been included in the methods session of the manuscript.

- In Figure 5, the authors investigate the cis-acting elements within the 5 UTR of SOX10 mRNA, involved in the HK2-mediated regulation, using a luciferase assay. They observed that deletions in the second half of the 5'UTR resulted in a dramatic reduction of Renilla luciferase expression, whereas deletions in the first half of the UTR did not significantly affect the translation of the construct. While most constructs did not exhibit significant changes in Renilla expression (Figure 5C), deletion number 4 and 5 appeared to cause a substantial reduction in Renilla mRNA abundance. To better assess the significance of this effect, it would be helpful if the authors provided p-values for the RNA expression data.
Since the enrichment of SOX10 mRNA is significantly reduced only in deletion number 4 (Figure 5D) in the HK2 IP experiments, could the authors speculate that this region is particularly crucial for the interaction? Additionally, can the authors explain why deletions 5 and 6, despite causing a drastic reduction in Renilla expression, appear to play a less evident role in the interaction?

The region corresponding to deletion 4 was predicted using Mfold (Zuker, Nucleic Acids Res., 2003) to form stem loops (A). Further experiments are required to elucidate whether the sequence or the secondary structure formed in this region is necessary for the interaction.

Regarding the luciferase assay, Figure 5B and 5C have been revised. The luciferase data (5B) is now presented as normalized to the RNA expression levels (5C) of the different vectors for clarity (B). We observed that, after normalization, only the deletion of region 4 significantly decreases Renilla expression. p-values were calculated by ordinary two-way ANOVA with Dunnett's multiple comparisons test (SD, n = 4) ($p \leq 0.05$). Although non-significant, deletion of the regions 5 and 6 decreases the Renilla expression. This decrease may be explained by disturbances in the secondary structures of SOX10 5'UTR or even in the activity of other trans-acting factors important for the translation of SOX10 mRNA beyond HK2.*

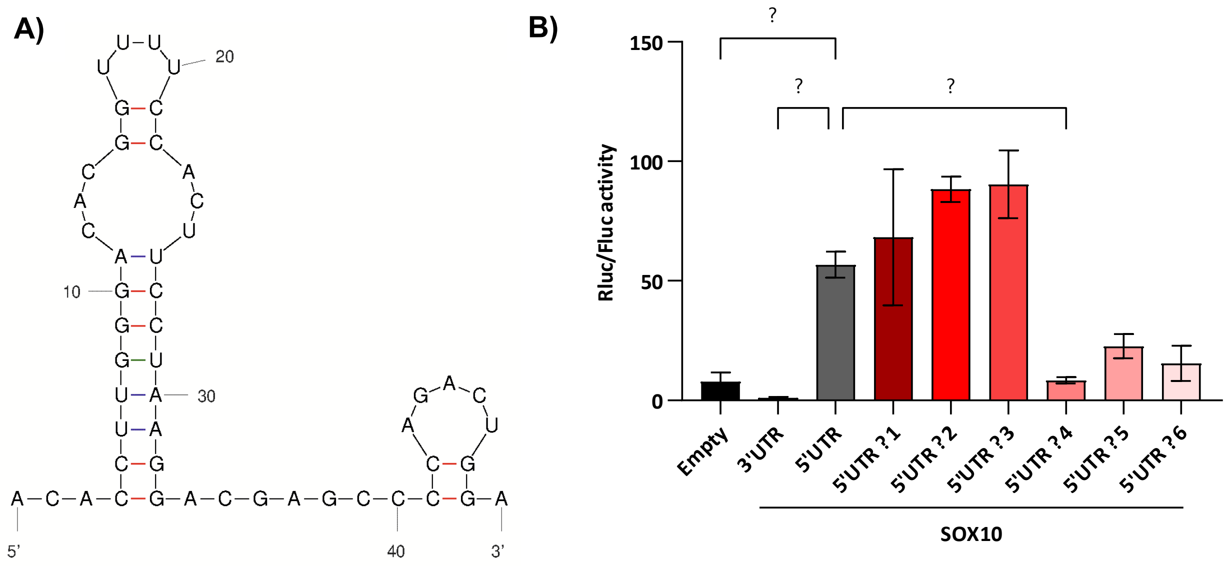

<u>Minor comments:</u>

- Introduction: Define GAPDH and PKM earlier in the text instead of line 6 in page 4

GAPDH and PKM have been defined earlier in the text. Instead of line 6 in page 4, GAPDH is now defined in line 18 in page 3, and PKM in line 23 in page 3.

- Introduction page 4 line 7: invert references Simsek, 2017 (for PKM) and Chang, 2013 (for GAPDH)

The references have been inverted as suggested.

- Figure 1: legend contains details of material and methods

The legend has been revised to remove the details of material and methods.

- For clarity, the confirmation of HK2-mediated translational regulation of SOX10 mRNA, demonstrated by monitoring SOX10 mRNA levels in each polysome fraction (Figure 2A), should be mentioned earlier in the text (after line 5). This would provide stronger justification for selecting this target, despite it not being statistically significant in Figure 1A.

As suggested, the confirmation of HK2-mediated translational regulation of SOX10 mRNA is now mentioned earlier in the text (after line 5) for clarity.

- Figure 3: indicate in the figure (or figure legend) concentration of RNase I used

Information regarding the RNase I concentration had been included in the methods session. However, this information has been included in the figure.

- Page 8, line 30: Figure S7A should be S6A

One additional figure was included as supplementary. This citation is now correct and corresponds to the mentioned results.

- Page 9, line 4: replace S6 with S6B

This citation has been revised in the manuscript as "Figure 4C and S7B" to correctly correspond to the mentioned results.

- Page 9, line 28: "Renilia" should be "Renilla"

"Renilia" has been correct to "Renilla" in the manuscript.

- Page 9, line 33: add reference to Figure 5B

The reference to Figure 5B has been removed from line 34 and will be added to line 31 instead, as follow:
"We observed a higher Rluc/Fluc ratio for the construct containing the 5'UTR compared to the 3'UTR or no UTR ("empty") ***(Figure 5B)*** *showing the importance of the 5'UTR in the translation of the SOX10 mRNA."*

- Figure S7 is cited as 'S7A' in the text before Figure S6, but S7 does not have multiple panels. It would be better to exchange the order of S6 and S7.

One additional figure was included as supplementary. This citation is now correct and corresponds to the mentioned results.

Reviewer #3

Major Comments

- The authors state that no changes in expression levels were detected between the different vectors (Figure 5C). Has this been statistically tested? Expression of delta4 and delta5 seems to be quite heavily reduced (ca 50%). Please comment on how this reduction would affect your quantification in Figure 5B. Is there a way to normalize the data to the respective expression levels?

RT-qPCR quantification of the Renilla mRNA was performed using the transfected cells in Figure 5B. The Renilla expression was normalized by the Firefly expression, and the data shown is relative to the expression of the Empty vector. The p-values were calculated by Kruskal-Wallis statistical test (SEM, n = 4 biological replicates).
Figure 5B and 5C, however, have been revised, and the luciferase data (5B) is now presented as normalized to the expression levels (5C) of the different vectors for clarity.

Minor Comments

- In Figure S1A the red numbers presumably represent quantification. The authors should include this in the figure legend.

This information has been included in the figure legend.

- It would be beneficial for the reader if Figure 1C would be split into two panels. One panel showing the 101 initially significant hits (e.g. as a volcano plot), followed by the current panel 1C. As the galactose condition is not introduced when Figure 1C is described, this can be confusing.

As suggested, the panel 1C has been split into 2 panels for clarity.

- Figure 1C: the cut offs used for analysis are missing in the figure legend.

This information has been added to the figure legend.

- Figure 1D: the number of biological replicates is missing in the figure legend.

This information has been added to the figure legend.

- Figure S2: the number of biological replicates is not clear. It should be at least >=3 and the SD should be shown.

The Seahorse metabolic flux assay is presented as a representative figure to confirm the metabolic switch of the melanoma cells from glycolysis to oxidative phosphorylation (OXPHOS) in A375 melanoma cells when cultured in galactose-containing medium instead of glucose. This metabolic shift has already been described and applied for glycolysis inhibition in different cell lines (Liu et al, 2022; Wang et al, 2018; Zhang et al, 2013; Weinberg et al, 2010).

This information has been added to the figure and figure legend.

- In Figure 3D it is stated that N=5, while for Figure S5 N=3. From my understanding the data is derived from the same experiments and thus the number of biological replicates should be similar.

The number of biological replicates has been corrected to N=4 in both figure legends.

- The authors state that HK2 is shifting to more dense fractions upon loss of RNA. While they briefly comment on this, it would be great for the reader if this finding could be further discussed.

Proteins exhibiting shifts on their distribution to denser fractions upon RNA loss have already been described in the R-Deep method (Caudron-Herger et al, 2019). In contrast to proteins found to shift to less dense fractions (i.e., HUR), dense-shifted proteins have no significant enrichment for domains linked to RNA-binding nor "RNA binding" molecular function (i.e., HK2). Dense-shifted proteins are then proposed to gain interaction partners upon loss of RNA, which could be due to the reduction of repulsive RNA charges or clearance of binding sites required for these interactions. These results may indicate that lighter-shifted proteins resemble more classical RBPs, while the other proteins may possess a wider spectrum of functions and domains.

This finding has been further discussed in the manuscript as follow (modifications are highlighted in bold):

*To further study HK2-RNA interaction, we subjected RNase-treated or untreated lysates of A375 cells to sucrose density gradient (5-25%) ultracentrifugation and fractionation. By these means, we found that 18% of the total cellular HK2 changed its distribution in the sucrose gradient following RNase treatment, indicating that the composition of HK2-containing complexes is partially dependent on RNA (*Figure 3D*). Intriguingly, complexes containing either GAPDH or PKM2 showed less sensitivity to RNase treatment* ***(Figure S6), therefore suggesting that HK2-containing complexes exhibit a greater dependence on RNA than complexes containing the other known RNA-binding glycolytic enzymes****. In contrast to HUR, HK2 presented an RNA-dependent shift to more dense fractions, thus suggesting that the presence of RNA might prevent the formation of heavy HK2-containing complexes.* ***HK2 is known to form complexes with different proteins (Thomas et al., 2022; Blaha et al., 2022; Guo et al., 2022) and directly interact with key substrates and metabolites, such as glucose and G-6-P. Notably, our CLIP assays revealed that neither HK2's interaction with VDAC1 at the outer mitochondrial membrane (OMM) nor glucose binding are required for its RNA-binding activity, supporting the hypothesis that RNA modulates HK2 complex formation.***

- For data presented in Figure 4 a second cell line is used. This is very helpful but should be briefly mentioned in the text as well.

The second cell line used in Figure 4 has been briefly mentioned in the text.

- Figure S4A is not readable. The color legend has been moved to the legend of Figure S4C, which is somewhat confusing.

The Figure S4A has been improved, and the legend revised.

- On page 8 line 30 there is a reference to Figure S7A. I guess it should read S6A.

One additional figure was included as supplementary. This citation is now correct and corresponds to the mentioned results.

- On page 9 line 4 there is a reference to Figure S6. I guess it should read S6B.

This citation has been revised in the manuscript as "Figure 4C and S7B" to correctly correspond to the mentioned results.

- The authors have to show the SD in Figure 5B, 5C and 5D instead of SEM (as they did in all the other figures).

The data in Figure 5 have been replaced to show SD instead of SEM as requested, and the legend has been updated.

- Figure S7 is a PCA which is only referenced in the methods part but not in the main text. Is this on purpose?

Yes, Figure S7 (now S8) is referenced in the methods session only.

Review COMMONS

4. Description of analyses that authors prefer not to carry out

Please include a point-by-point response explaining why some of the requested data or additional analyses might not be necessary or cannot be provided within the scope of a revision. This can be due to time or resource limitations or in case of disagreement about the necessity of such additional data given the scope of the study. Please leave empty if not applicable.

Reviewer #2

- In the final part of the paper, the authors evaluate the role of HK2-mediated regulation of SOX10 mRNA in the colony formation and proliferation of melanoma cells. In the rescue of KH2 depletion by ectopic expression of SOX10 mRNA, the comparison should be performed between the "Empy" and "SOX10". Evaluation of the rescue experiment should include as a control the ectopic expression of SOX10 ORF with the WT 5'UTR and with the 5'UTR harboring the deletion number 4 (i.e. one deletion showing reduce luminescence signal and lower enrichment in IP).

Indeed, our results from luciferase assays coupled with RT-qPCR experiments indicate that region 4 (152- 201 nts) is crucial for driving SOX10 5'UTR-mediated translation. Therefore, a rescue experiment using ectopic expression of SOX10 ORF with the 5' UTR harboring the deletion number 4 may not be possible due to inefficient translation/expression of the transduced ORF.

Reviewer #3

- The authors show that Sox10 mRNA shifts to lighter polysome fractions upon depletion of HK2. Could the authors please use PKM2 and GAPDH KD cells and perform a similar experiment to prove that this shift is directly connected to loss of HK2 and not secondary?

In agreement with our polysome data suggesting that HK2 KD leads to a decrease in the efficiency of SOX10 mRNA translation, we showed by western blot that HK2 KD leads to a decrease in SOX10 protein levels (and not in the RNA level). As mentioned in the manuscript, this effect seems to be specific to the loss of HK2, since GAPDH KD did not impact SOX10 expression (both protein and RNA levels). These results suggest that the execution of the polysome profiling upon GAPDH/PKM2 KD is unlikely to provide significant additional contributions to the findings of the manuscript.
