# Supplementary material for: Hexokinase 2 is an RNA-binding protein that regulates mRNA translation independently of glycolysis and induces melanoma cell proliferation
Source: PLoS Biol. 2025 Sep 16;23(9):e3003364. doi: 10.1371/journal.pbio.3003364 (PMC12494293; doi:10.1371/journal.pbio.3003364)
Supplement: Attachment — Submitted filename: 2025-07-16_LB_revision_answers-Ana.docx [file pbio.3003364.s035.docx]

**General Evaluation**

This manuscript explores a novel, non-metabolic role of Hexokinase 2 (HK2) as an RNA-binding protein (RBP) that regulates mRNA translation in melanoma cells. While the metabolic functions of glycolytic enzymes in cancer are well established, their emerging roles in translational control are less well defined. The authors present compelling evidence that HK2 directly binds to the 5′ UTR of SOX10 mRNA and promotes its translation independently of its enzymatic activity and metabolic function. These findings highlight a previously unrecognized function of HK2 in tumor cell proliferation and offer valuable insights into the interplay between metabolism and gene expression regulation in cancer.

The authors have addressed all my major concerns raised in the first round of revision. The manuscript is now much improved, with the inclusion of key experiments that convincingly support the role of HK2 as a regulator of SOX10 mRNA translation.

**Comments**

- While I understand the rationale for focusing on the selected targets, the purpose of the translatome and RNA-seq analyses remains somewhat unclear and not connected with the rest of the paper. Since the six targets of interest were ultimately identified by another strategy, it would be helpful to specify whether they were absent from the RNA-seq data due to a lack of statistical significance. If so, please indicate in page 12, line 9 the p-values for those genes to show how close they were to the significance threshold (e.g., p < 0.05).

As mentioned in the text, “Depletion of HK2 led to a significant decrease in the abundance of 3 of them (*SOX10, CDH2, and WNT5A*) in RNAs extracted from polysome fractions, but not in total RNAs (Fig. 1F). Of note, these mRNAs were not found in the list of 101 candidate mRNAs regulated at the translation level by HK2 (Table S1) because of a lack of statistical significance (*p*>0.05).”

The following text has been included in the legend of Fig. 1F as “HK2 depletion significantly decreased the abundance of *SOX10, CDH2,* and *WNT5A* mRNAs extracted from polysome fractions, but not in total RNAs. Of note, these mRNAs were not found in the translatome data as regulated by HK2 because of a lack of statistical significance (*SOX10*, *p* = 0.3; *OCLN*, p = 0.4; *WNT5A*, p = 0.7).”

- Figure 1F: For the qPCR results, please avoid setting the control arbitrarily to 1. Use proper normalization, as done for the Western blot quantification.

The controls were arbitrarily set to 1 in order to represent the mRNAs abundance in both total RNA (input) and polysomal RNA in the same scale. Some of the mRNAs tested and presented are less abundant in polysome fractions than input, as exemplified bellow with the *CDH2* mRNA (left: data presented in figure 1F where we set the controls to 1; right: representation of the data presented in figure 1F without setting the controls to 1):

Therefore, we would rather present the data as setting the controls arbitrarily to 1.

- Figure 2A: Please include statistical analysis for the different fractions.

Statistical information was included. p-values were calculated by unpaired, two-tailed Student’s t-test test (SD, n = 3 biological replicates) and significant comparisons are shown (* p ≤ 0.05).

- Figure 6E: Statistical information is missing in the plot. Please include it for completeness.

Statistical information was included.

- Regarding the rescue experiment: I originally suggested including ectopic expression of SOX10 ORF with both the WT 5′ UTR and the 5′ UTR harboring deletion #4 (i.e., the deletion that showed reduced luciferase activity and lower enrichment in IP).

The authors respond that this experiment is not feasible due to inefficient translation of the transduced ORF with the deleted 5′ UTR. My original point was that such a control would be critical to demonstrate that the deleted region is indeed necessary for regulation (i.e. no rescue should be observed when using that construct). That said, I acknowledge that this conclusion is already well supported by the other complementary approaches.

**Minor Comments**

- Abstract, lines 25–26: The sentence "by promoting the release of HK2..." is unclear. Please clarify—do high glucose conditions promote HK2 release from the outer mitochondrial membrane (OMM)? Rephrasing is needed for clarity.

This sentence was modified to bring more clarity to the text, and it has been replaced for:
“(…) we found that high glucose conditions, which promotes the release of HK2 from the OMM, induces an increase in HK2-*SOX10* mRNA interaction and *SOX10* mRNA translation in the cytoplasm.”

- Introduction, page 7, line 31: Please define or briefly explain SNAIL-mediated EMT for clarity.

The sentence was replaced by: “(…) HK2 contributes to epithelial-mesenchymal transition (EMT) and metastasis in breast cancer via the transcription factor SNAIL, a key EMT inducer.”

- Introduction, page 8, line 6: Spell out and define "OXPHOS" at first mention.

OXPHOS was first mentioned and defined in page 3, lines 3 and 4.

“(…) even when local conditions are suitable to utilize the oxidative phosphorylation (OXPHOS), a more efficient mechanism to generate ATP.”

- Page 14, lines 33–35: Rephrase this sentence. It is currently unclear and appears to misuse “either…or.”

We removed “either” from the sentence for clarity.

- Page 28, lines 16–18: Add a reference to the relevant figure.

The reference was added in page 28, line 11.
